# Quantitatively analyzing the relationship between non-pharmaceutical interventions and the direction of virus evolution using a dynamic model

**DOI:** 10.3389/fpubh.2025.1542759

**Published:** 2025-05-09

**Authors:** Zuiyuan Guo, Yuheng Chen, Hongbo Liu, Guangquan Xiao, Di Yu, Zhaojia Zhang, Yimin Yang, Zhongwei Yin, Huibin Zhang

**Affiliations:** ^1^The First Department of Infectious Disease Prevention and Control, Center for Disease Control and Prevention in Northern Theater Command, Shenyang, China; ^2^College of Communication Engineering, Jilin University, Changchun, China; ^3^Department of Information, Center for Disease Control and Prevention in Northern Theater Command, Shenyang, China; ^4^Department of Comprehensive Planning, Center for Disease Control and Prevention in Northern Theater Command, Shenyang, China; ^5^Department of Critical Care Medicine, The First Hospital of Jilin University, Changchun, China; ^6^Department of Medical Protection and Military Operational Medicine, Center for Disease Control and Prevention in Northern Theater Command, Shenyang, China; ^7^Department of Medical Protection, Center for Disease Control and Prevention in Northern Theater Command, Shenyang, China

**Keywords:** SARS-CoV-2, mutation, virus evolution, social distance, genetic algorithm, dynamic model

## Abstract

**Introduction:**

Since the emergence of COVID-19 in 2019, SARS-CoV-2 has persisted in mutating, giving rise to multiple variants of concern that have triggered several pandemics globally. The evolutionary trajectory of the virus is shaped by a combination of stochastic factors and non-pharmaceutical interventions (NPIs). Investigating the direction of virus evolution and its underlying determinants is crucial for forecasting epidemic trends and formulating scientific responses to emerging infectious diseases.

**Methods:**

To delve into the intricate relationship between NPIs and the virus’s transmissibility, virulence, and immune evasion capabilities, as well as to explore the sociological mechanisms driving virus evolution, we developed a genetic algorithm grounded in a population dynamics model. This model simulates the processes of virus mutation and epidemic dissemination, enabling us to analyze the correlation between intervention strategies and the evolutionary path of the virus.

**Results:**

Our study reveals that, under the influence of NPIs, dominant strains capable of widespread transmission within the population exhibit substantially elevated immune evasion capabilities and heightened infectivity. Notably, the evolution of virulence did not display a discernible trend, aligning with the observed epidemic characteristics of COVID-19. It was found that the stricter the implementation of NPIs, the more favorable the conditions for rapidly and thoroughly containing virus transmission and mutation. Conversely, the relaxation of these measures may pose a risk of recurring epidemics fueled by continuous viral mutations.

**Discussion:**

Presently, the potential emergence and widespread transmission of SARS-CoV-2 variants with increased virulence cannot be discounted. Therefore, it is imperative to continuously monitor the dynamic shifts in the epidemic landscape and the antigenic variations of new variants. Simultaneously, it is necessary to devise and prepare prevention and control strategies to effectively manage outbreaks caused by highly pathogenic variants.

## 1 Introduction

SARS-CoV-2 continues to evolve. Since the onset of the COVID-19 pandemic, the World Health Organization (WHO) has identified multiple variants of concern (VOCs), based on their potential for increased transmission, replacement of existing strains, causing new infection waves, and necessitating adjustments in public health responses (1). Coronaviruses exhibit high recombination rates, insertions and deletions, and point mutations, although the rates are lower than for other RNA viruses due to the proofreading, leading to a plethora of variants during replication (2). Most variants have minimal impact on the virus’s characteristics. Nonetheless, some can significantly alter infectivity, disease severity, immune escape capabilities, the efficacy of treatments, diagnostics, and the effectiveness of public health and social measures (3). As of December 11, 2021, the WHO has recognized five VOCs since the pandemic’s start: Alpha, Beta, Gamma, Delta, and Omicron (4).

NPIs have proven to be effective tools in mitigating the spread of pandemics and have played a pivotal role in the management of the COVID-19 outbreak (5). In March 2020, the emergence of the wildtype strain triggered the initial wave of the epidemic in England. As the virus rapidly disseminated throughout the population, leading to a substantial surge in hospitalizations and mortalities, the government implemented NPIs to promote social distancing, with the aim of curtailing the rise in severe cases and alleviating the burden on the healthcare system (6). These measures were lifted after effectively reducing hospitalizations and mortalities, and social distancing returned to normal. However, the advent of the Alpha variant precipitated a second wave of infections in England from September 2020 to April 2021, during which the government twice reinstated NPIs to control virus transmission (6). From December 2020 onwards, the rollout of vaccination programs in the UK bolstered population immunity, thereby limiting the dissemination of both the Alpha and Delta variants. Consequently, in the summer of 2021, NPIs were gradually lifted in accordance with the national policy outlined in the Roadmap out of lockdown (7, 8). Nevertheless, in response to the rapid spread of the highly transmissible and immune-evasive Omicron variant, the UK introduced Plan B in December 2021 to contain the exponential rise in cases (9). The UK’s pandemic response underscores how novel strains can evade the population’s immune memory established against previous strains, becoming dominant and fueling new transmission chains, thereby compelling governments to reimplement public health measures to counteract successive waves of the epidemic in a recurring cycle.

The epidemic prevention model employed in the UK exhibits a significant level of representativeness on a global scale, characterized by the cyclic appearance of virus variants and the corresponding implementation of targeted prevention measures. This intriguing phenomenon has sparked our curiosity, prompting us to delve into its underlying mechanisms and endeavor to elucidate the intricate relationship between human behavior and viral evolution. Observations have shown that VOCs of SARS-CoV-2 have led to multiple reinfections within the population (10, 11), with the Omicron variant demonstrating heightened infectivity and immune evasion capabilities (12–14), while the severity of illness appears to be reduced (15). However, the extent to which these phenomena are influenced by NPIs remains scientifically unsubstantiated. To tackle these questions, we developed a genetic algorithm and utilized computer simulations to mimic the entire trajectory of virus mutation, epidemic dissemination, and NPI implementation. This approach allowed us to conduct an in-depth analysis of the correlation between fluctuations in social distancing and the direction of viral evolution. We anticipate that this research will provide a robust theoretical foundation for exploring the mechanisms by which NPIs influence viral evolution and aid in predicting the evolutionary path of SARS-CoV-2 and potential future unknown infectious diseases.

The academic community has launched multiple scientific investigations to explore the trajectory of viral evolution. For instance, Sunagawa J employed longitudinal viral load data to demonstrate that isolation can exert a selective pressure, favoring viruses with an earlier and higher peak viral load but a shorter duration, in the evolutionary pathway of SARS-CoV-2 (16). Woo HJ presented a quantitative model to simulate the evolution of simian immunodeficiency virus under the pressure exerted by the cytotoxic T-lymphocyte immune response, thereby delineating the evolutionary direction of the virus under immune selection (17). Han WK developed a machine learning-driven antigen evolution prediction model, which, from a molecular biology perspective, provided evidence that the predicted SARS-CoV-2 variants exhibit heightened immune evasion capabilities (18). These studies, originating from diverse disciplines, delve into the biological and sociological mechanisms underlying viral evolution, laying a robust scientific foundation for a comprehensive understanding of the factors that shape viral evolution. However, there remains a notable gap in the availability of an infectious disease dynamics model that integrates population, individual, and viral dynamics to simulate the entire process of virus mutation and epidemic spread under the influence of NPIs, and to elucidate the mechanism by which these measures exert natural selection on viral variants.

We have developed a dynamic model that centers on individuals, utilizing the concept of genetic algorithms to simulate the intricate and reciprocally influential patterns of virus mutation, transmission, and the implementation of NPIs in real-world scenarios. By monitoring the progression of epidemics and the trends in strain evolution, and by analyzing the underlying mechanisms through which epidemic intervention measures influence strain evolution, our model aims to provide a theoretical foundation for predicting the evolutionary trajectories of SARS-CoV-2, influenza, and other respiratory viruses characterized by high mutation rates.

## 2 Materials and methods

The study revealed that, among the 6,068 NPIs implemented during March–April 2020, when the majority of European countries and US states experienced their initial waves of infections, the most effective measures encompassed the cancelation of small gatherings, closure of educational institutions, imposition of border restrictions, enhancement of personal protective equipment availability, and restrictions on individual movements (5). These interventions can be collectively characterized as strategies aimed at minimizing virus exposure opportunities by promoting social distancing or reducing overall social activity levels. In our model, we converted the efficacy of NPIs implementation into variations in social activity levels, denoted as *β* (*t*), ranging from 0 to 1. Here, 0 signifies a complete halt of interpersonal contact and social activities due to interventions, and 1 represents the normal level of social activity in the absence of any interventions. This parameter was subsequently used to adjust the effective reproduction number (*Rt*), thereby influencing the rate of virus transmission. Additionally, we employed SARS-CoV-2 as a case study for epidemic simulation, whereby certain model parameters were extracted from COVID-19 data, while others, not readily available from existing literature or surveys, were estimated based on previous research and epidemiological practice.

### 2.1 The premises of the model

**Figure 1:**
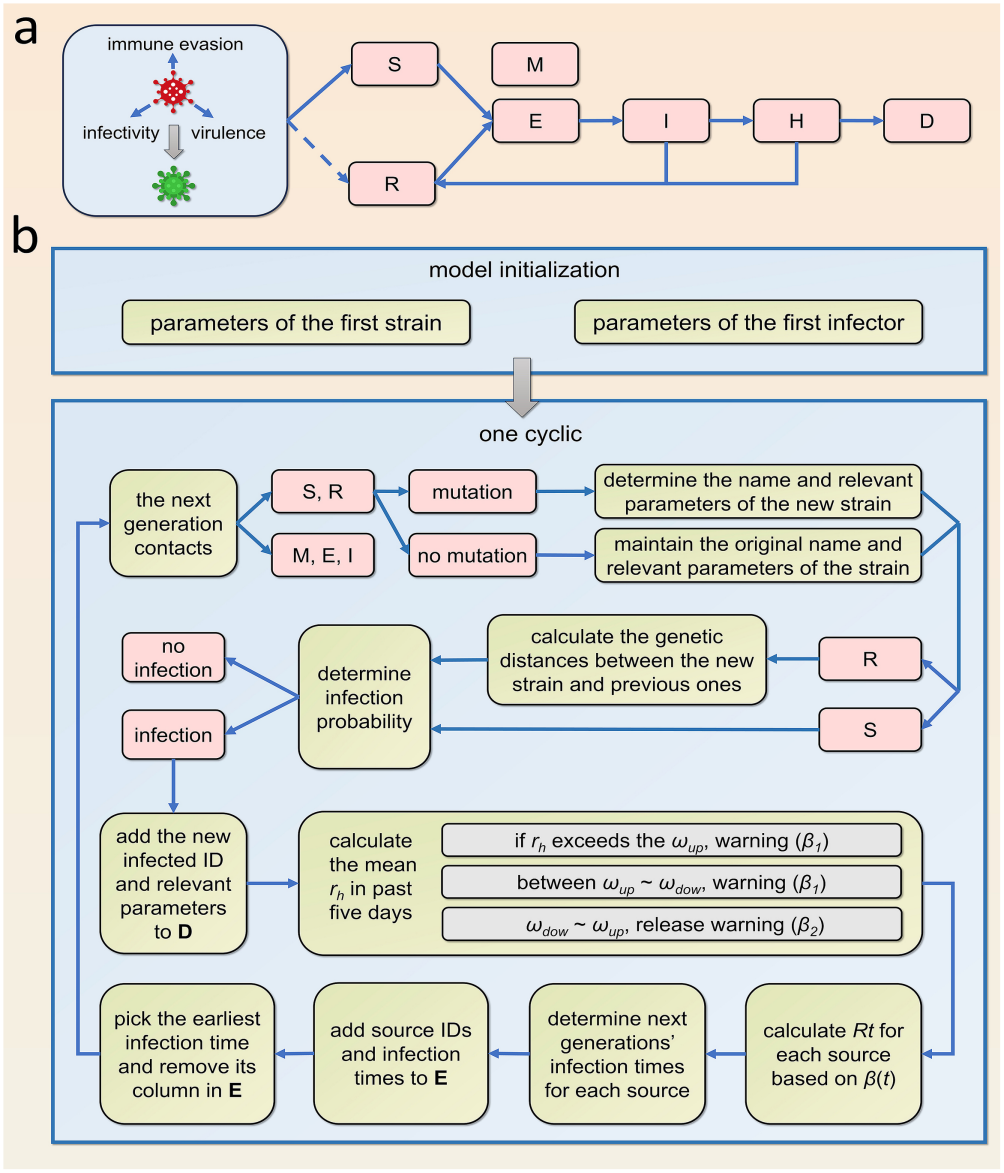
Model schematic and flowchart. **(a)** The transformation relationship of populations in different infection statuses, where S, E, I, H, R, D, and M represent susceptible, exposed (latent infection), infectious (mild and pre-hospitalized severe cases), hospitalized, recovered, deceased individuals, and those with innate immunity, respectively. Solid lines indicate definitive transitions, while dashed lines represent probabilistic transitions. **(b)** The flowchart for the model’s design, where **D** and **E** represent the information data frame and the time data frame, respectively.

**Figure 2:**
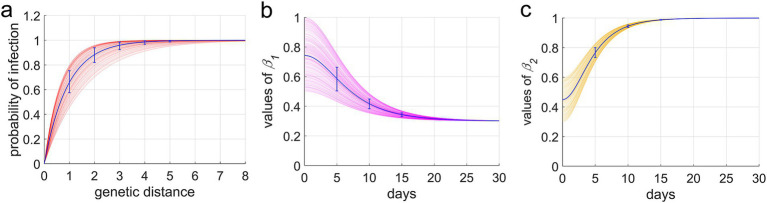
Infection rates in recovered individuals and the dynamics of social activity levels: decreases and recovery functions. **(a)** The functional relationship between the probability of reinfection in recovered individuals and genetic distance. **(b,c)** Represent the functions of social activity levels decreasing *β*_1_(*t*) and increasing *β*_2_(*t*) from different levels, respectively.

To clarify our research theme, we simplified the model structure and established the following premises:

(1) The epidemic unfolds within a closed town with a fixed population of 20,000, disregarding births, natural deaths, and migration.
(2) Given that different age groups exhibit varying rates of severe illness upon SARS-CoV-2 infection (15), we categorized the population based on the age structure prevalent in China.
(3) During viral replication, the genome encoding the spike protein has a certain probability of undergoing recombination and errors, representing the mutation rate (*q*). This rate signifies the likelihood of variants emerging when the virus is transmitted from an infector to second-generation infectees. Changes in the spike protein structure can randomly alter the virus’s infectivity, virulence, and immune evasion capabilities. Consequently, we stipulate that the antigenic properties of mutant strains must differ from their parent strain by at least a factor of *p* in these three dimensions.
(4) The population is stratified according to infection status into susceptible (S), exposed (E) (during the incubation period), infectious (I), hospitalized (H), deceased (D), and recovered (R) individuals. Additionally, recognizing that a small portion of the population possesses innate immunity (19), individuals who cannot be infected by any strain (M) were included. The transition relationships among these groups are illustrated in Figure 1a. I can only infect S and R. Effective contact between I and S results in S becoming infected, while the probability of R becoming reinfected upon contact with I depends on the genetic distance between the strains they were previously infected with. A greater genetic distance, indicating larger antigenic differences, correlates with a higher probability of *R* being reinfected. We define the relationship between the infection probability (*p_inf_*) and genetic distance (*d*) using Equation 1 (function depicted in Figure 2a).

(1) $$p_{\inf}=1‐\exp(‐\mathrm{\lambda d})$$

Where *λ* is the immune escape index.

**Figure 3:**
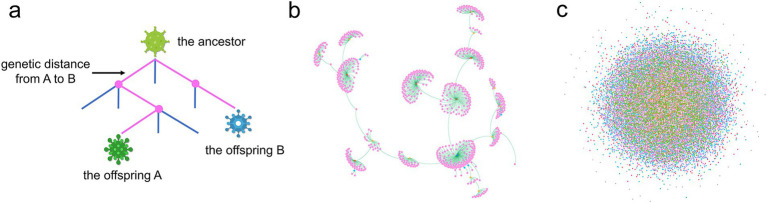
Genetic distance, phylogenetic tree, and transmission chains. **(a)** The genetic distance in the phylogenetic tree between offspring A and B. The purple path indicates the genetic distance between the two progeny strains, revealing that their genomes, coding for the spike protein, have five different mutation sites. **(b)** The phylogenetic tree reflecting the process of virus evolution. Each strain is represented by a dot, with varying colors indicating the number of infections. Each mutation in the virus adds a branch for a new progeny. **(c)** The transmission chains of the epidemic. Each dot represents an infected individual, and each line represents a transmission relationship. The color of the dot indicates the number of times the individual was infected.

(5) The infectivity, virulence, and immune evasion capabilities of the virus are primarily governed by the structure of the spike protein on its surface (20). Genetic distance serves as a metric to quantify the number of differences in the genome sequences encoding this spike protein. This distance is assessed by computing the distance between the branches of the evolutionary tree to which each strain belongs (Figure 3a).
(6) The virulence of the virus is gauged by the hospitalization rate *(rh)*. The implementation of NPIs depends on the hospitalization rate of newly diagnosed patients within the last 5 days, which is calculated as the number of new hospital admissions divided by the total number of newly infected individuals. When this rate surpasses a predetermined upper threshold *(ωup)*, both the government and the public are prone to enhance social distancing, resulting in a diminution of social activity levels. Conversely, when the hospitalization rate declines to a lower threshold *(ωdow)*, social distancing measures are eased, leading to an elevation in social activity levels. Given the impossibility of completely eliminating human contact, social activity levels will not plummet to zero but will tend to stabilize at a minimum value, denoted as *βmin*. The variables *β1(t)* and *β2(t)* embody the functions describing the decrease and increase in social activity levels, respectively, as illustrated in Equation 2. The graphical representations of these functions are provided in Figures 2b,c. Definitions and values for all parameters incorporated in the model are furnished in Table 1.

(2) $$\begin{array}{l} \beta_{1}(t)=2[\beta (t_{0})-\beta_{\min}]/[e^{-\gamma_{\mathrm{dow}}(t-t_{0})}+e^{\gamma_{\mathrm{dow}}(t-t_{0})}]+\beta_{\min}\\ \beta_{2}(t)=1-2[1-\beta (t_{0})]/[e^{-\gamma_{\mathrm{up}}(t-t_{0})}+e^{\gamma_{\mathrm{up}}(t-t_{0})}] \end{array}$$

**Table 1:** Model parameters.

Description	Distribution characteristics	Numerical values	Sources
Total population	20,000	constant	assumed
The population proportions for the age groups 0–24, 25–44, 45–64, and 65 + years, respectively	0.276, 0.284, 0.292, 0.148	constant	(36)
The hospitalization rates, *r_h_*, for various age groups	0.0033, 0.0145, 0.0331, 0.0789	constant	(15)
The basic reproduction number *R*0	2–5	uniform distribution	assumed
immune escape index *λ*	0.6–1.6	uniform distribution	assumed
The proportion of the population possessing innate immunity *p_imm_*	0.1	constant	assumed
The incubation period (days)	*μ* = 3.1 *σ* = 2.6	normal distribution	(37)
The rate of strain mutation *q*	0.001, 0.002, 0.003	constant	assumed
genetic variability *p*	0.1	constant	assumed
mortality rate of inpatients *δ*	0.0467	constant	(15)
the rate of decline in social activity *γ_dow_*	0.2	constant	assumed
the rate of recovery in social activity *γ_up_*	0.3	constant	assumed
The lowest point of decrease in social activity *β_min_*	0.1, 0.2, 0.3	constant	assumed
The threshold at which an increase in the hospitalization rate triggers a decline in social activity *ω_up_*	0.05	constant	assumed
The threshold at which a decline in the hospitalization rate triggers an increase in social activity *ω_dow_*	0.04	constant	assumed
The duration from symptom onset to hospitalization in non-admitted patients.	2–7 days	uniform distribution	(38)
The duration from symptom onset to hospitalization in admitted patients.	4–12 days	uniform distribution	(39)

### 2.2 The construction process of the model

The construction process of the model is detailed as follows: Based on the aforementioned assumptions, we leveraged the MATLAB language to develop a program implementing a genetic algorithm. By iteratively executing this program, the evolutionary trajectory of the virus was simulated. The specific methodology employed for constructing the model is outlined below.

(1) Initially, the program is initialized, which includes setting up variables, parameters, age groups, IDs for the innately immune population, and the antigenicity of the initial virus strain. Additionally, certain epidemiological characteristics of the index case need to be defined, such as his ID, the strain number of the infection, the number of infections (here it is set to 1), age, time of infection, onset time, admission time, discharge time, and current infection status (including infected, recovered, dead), etc. These data are stored row-wise in a data frame, designated as the information data frame. Based on the value range provided in Table 1, the basic reproduction number (*R0*) for the index case is randomly generated. Using *R0* as the mean, a Poisson-distributed random number is generated to represent the number of second-generation infectees originating from the index case. Then, *n* time points are randomly selected from within the infectious period according to a uniform distribution, serving as the infection times for these infectees. Subsequently, the source ID and the infection time of each infectee are stored in a data frame, designated as the time data frame, where each column represents a transmission relationship between an infector and an infectee. The program code for this is found in Appendix I, lines: 1–125.
(2) After completing the initialization, simulate the ongoing spread of the virus through time iteration. First, select the earliest infection time in the time data frame, extract the corresponding column and record the ID of the infectious source, then remove this column from the data frame. Subsequently, update the infection status of all infected individuals at that time in the information data frame. The population that the infectious source can effectively contact includes: S, E, I, R, and M. Randomly select one of these individuals as the contact. Infection can only occur if the contact is S or R. Before determining the newly infected individual, it is necessary to assess whether a mutation occurs during transmission based on the mutation rate. If a mutation occurs, first name the new virus strain and then randomly set its infectivity, virulence, and immune escape capability.

If the randomly selected contact is S, the probability of infection is 1. If the contact is R, since R has previously been infected with several virus strains and has developed specific antibodies against each of these strains, the new virus strain can only cause infection by evading recognition by all antibodies. The infection probability, *q_inf_*, is calculated by measuring the genetic distance between the new virus strain and the *i*th previously infecting strain of R, and then applying Equation 1 to obtain the infection probability for that particular strain. Finally, the infection probabilities for all previously infecting strains of R are multiplied together, i.e., $p_{\inf}^{1}p_{\inf}^{2}\cdots p_{\inf}^{n}$ , to obtain *q_inf_*. A Bernoulli random number is then generated based on *q_inf_*, where 0 indicates no infection and 1 indicates infection. If infection occurs, the epidemiological characteristics of the secondary infected individual need to be determined, following the details outlined in Step (1), and this information is appended as a new row to the information data frame. The corresponding program code can be found in Appendix I, lines 127–330.

(3) After identifying the secondary infected individuals, calculate the social activity level *β(t)* at time *t*. First, calculate the new hospitalization rate over the past 5 days. When the new hospitalization rate exceeds ωup, it indicates that the current virus epidemic poses a serious threat to public health, prompting the government to implement NPIs to increase social distancing. These measures will lead to a decrease in social activity level and slow down the spread of the virus. When the new hospitalization rate drops to ωdow, it signifies that the epidemic’s threat has diminished. Considering economic and other factors, the government will lift the restrictions on social distancing, leading to an increase in social activity level. However, this increases the risk of the virus resuming transmission among the population and potentially causing a resurgence of the epidemic. The code for this part of the program can be found in Appendix I, lines 332–379.
(4) Once *β(t)* is determined, we proceed to calculate the number and timing of secondary infectees transmitted by each infectors at time *t*. Firstly, we identify all infectors who are in the infectious period at time *t*. Then, for each infectious individual, we calculate the *R(t)* based on the strain they are infected with. *R(t)* is positively correlated with *R0*, the proportion of active individuals in the population, and *β(t)*. After obtaining *R(t)*, we randomly generate the number and infection times of the next generation of infectees according to a Poisson distribution with *R(t)* as the mean. The infectious source IDs and corresponding infection times are stored in the time data frame in columns. This completes one iteration of time. After tens of thousands of iterations, the loop terminates when no secondary infections are generated or the termination time is reached, marking the end of one epidemic simulation. The code for this part of the program is found in Appendix I, lines 381–442. The design framework of the model is shown in Figure 1b.

Even though we did not use differential equations to construct the model, to clearly express the quantitative relationships between different population groups, we employ a set of equations as follows:


(3) $$\begin{array}{l} S^{'}=-\sum_{i}\delta_{i}\beta (t)\theta (t)I_{i}S/N\\ E^{'}=\sum_{i}\delta_{i}\beta (t)\theta (t)I_{i}S/N+\sum_{i}\delta_{i}\beta (t)\theta (t)q_{\inf}^{i}I_{i}R/N-\mathrm{\varphi E}\\ I^{'}=\mathrm{\varphi E}-\mu_{m}p_{m}I-\mu_{h}(1-p_{m})I\\ H^{'}=\mu_{h}(1-p_{m})I-\mathrm{h\sigma H}-h(1-\sigma )H\\ R^{'}=\mu_{m}p_{m}I+\mathrm{h\sigma H}-\sum_{i}\delta_{i}\beta (t)\theta (t)q_{\inf}^{i}I_{i}R/N\\ D^{'}=h(1-\sigma )H \end{array}$$

In Equation 3


$$\begin{array}{l} N=S+E+I+R+M\\ \theta (t)=N/(N+H+D) \end{array}$$

In Equation 3, *N* and *θ*(*t*) represent the total number of people active in society at time *t* and their proportion of the total population, respectively. *β*(*t*) indicates the social activity level at time *t*, with the calculation method detailed in Equation 2. *δ_i_* refers to the effective contact rate of individuals infected with the *i*-th strain (i.e., the number of effective contacts a source of infection makes per unit of time at the beginning of an outbreak). 1/*φ* represents the incubation period. $q_{\inf}^{i}$ indicates the probability of a recovered individual being infected by strain *i*, *p_m_* is the proportion of patients with mild symptoms, 1/*μ_m_* and 1/*μ_h_*, respectively, represent the infectious period of mild (from onset to recovery) and severe cases (from onset to hospitalization), and 1/*h* and *σ* denote the hospitalization period for severe cases and the recovery rate, respectively.

### 2.3 Sensitivity analysis

Given the theoretical nature of this epidemiological study, where predefined conditions may not directly correspond with reality, fitting the model to actual survey data presents challenges. To validate the scientific and robust nature of the research method, the study utilized the PRCCs and Latin Hypercube Sampling method for sensitivity analysis. This widely applied algorithm calculates correlations between a set of parameters and model outputs, excluding the linear effects of the targeted parameter (21). The parameter space was segmented into equal intervals, with one sample randomly chosen from each. These samples were then integrated into the model to determine outputs at various time points, resulting in a series of standard coefficients that illustrate the correlation between each parameter and the model output (21, 22). Further details on sensitivity analysis are available in Appendix II. All methodological analyses were conducted using MATLAB R2019a software (MathWorks, Natick, Massachusetts, USA).

## 3 Results

### 3.1 Phylogenetic tree and transmission chains

The simulation of a full-scale epidemic outbreak yielded a phylogenetic tree illustrating the evolution of the virus. As depicted in Figure 3b, this tree encompasses 810 strains spanning seven generations, with the descendants of the original strain being the most numerous, totaling 139. Out of all the strains, six were implicated in infections affecting over 10,000 individuals accounting for less than 1%, and 17 strains led to more than 1,000 infections, collectively accounting for approximately 2% of the total. Strains that infected fewer than 100 individuals constituted 97% of the total, with those infecting fewer than 10 individuals comprising 92%. The tree visually demonstrates that strains causing larger outbreaks tend to produce more progeny; however, among these progenies, only a very small proportion have the potential to cause a pandemic, while the majority do not continue to replicate and disseminate significantly.

Figure 3c presents the transmission chain of the epidemic. Due to the large number of infections, it is not possible to clearly distinguish the transmission relationships between the initial infectors and the subsequent infectees. However, the distribution of the number of infections can be discerned by color, with a higher proportion of individuals having experienced 5 to 8 infections.

### 3.2 Influence of antigenic characteristics on the spread of strains

**Figure 4:**
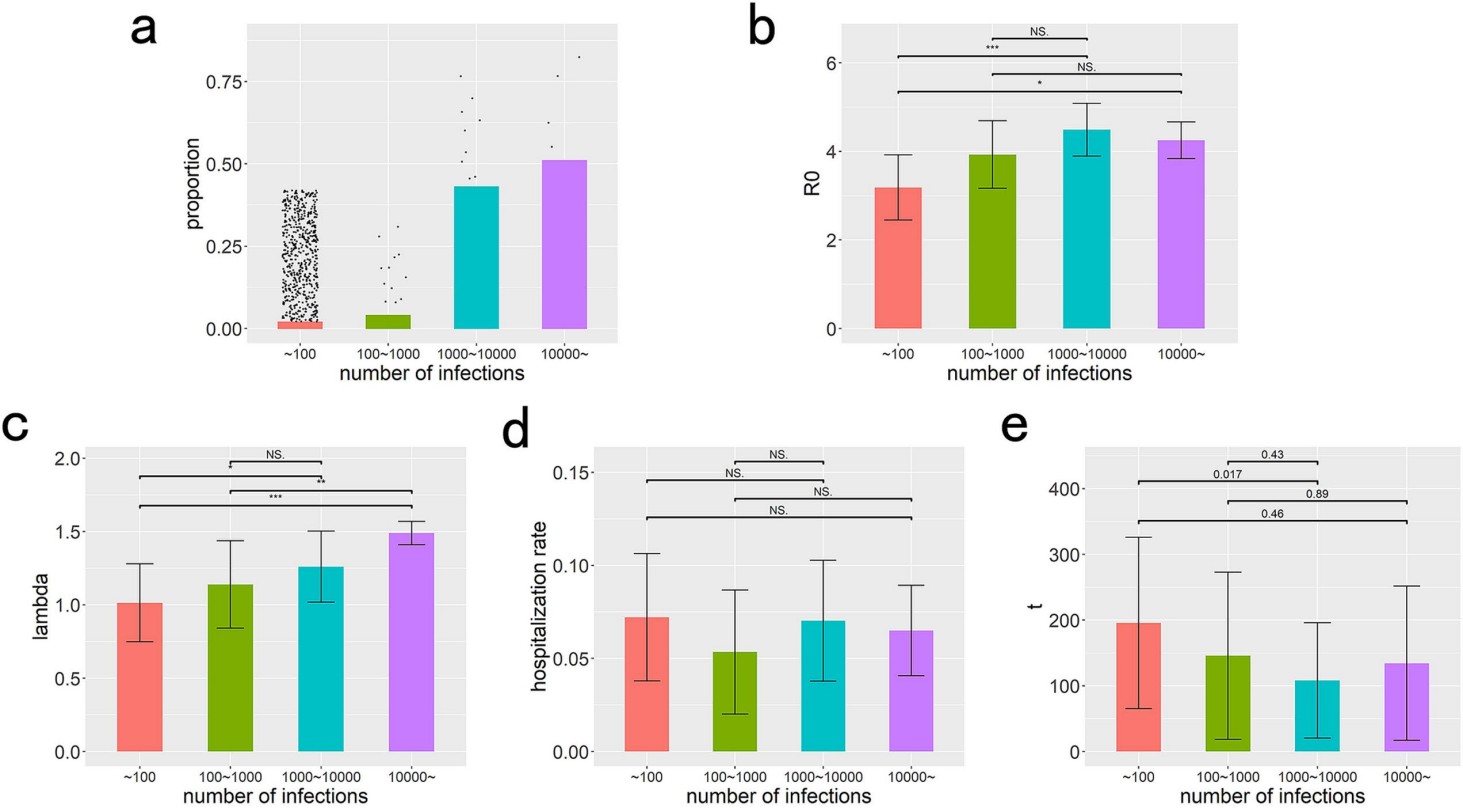
Distribution characteristics and influencing factors of variant spread scale. **(a)** Distribution characteristics of variant spread scale. The scatter plot shows the distribution of the number of variants causing different scales of infection, while the bar chart represents the proportion of infections caused by variants of different scales to the total number of infections. **(b–e)** Show the median and interquartile range for the *R0*, immune escape index, hospitalization rate, and emergence time of strains with different infection scales, along with their statistical test results. NS, *, **, *** represent the results of statistical tests as not significant, *p* < 0.05, *p* < 0.01, and *p* < 0.001, respectively.

Based on the phylogenetic tree, we can conduct a more quantitative and in-depth analysis of the influence of a strain’s antigenic properties on its dissemination. Figure 4a reveals that the proportions of strains causing infections in more than 10,000, 1,000 to 10,000, 100 to 1,000, and fewer than 100 individuals are 0.7, 1.5, 2.0, and 96%, respectively. In contrast, their corresponding proportions in terms of the total number of infections are 51, 43, 4.0, and 2.0%. This disparity indicates that a minuscule fraction of strains has the potential to trigger large-scale epidemics, whereas the majority of strains are unable to sustain significant transmission.

In Figures 4b–e, we conducted a comparative analysis of the statistical differences in the basic reproduction number (*R0*), immune escape index, hospitalization rate (serving as a proxy for virulence), and emergence time across four distinct groups of strains, categorized by their infection scales. The statistical results revealed that the disparities in the immune escape index were the most pronounced, with *R0* exhibiting the second most significant differences. Notably, no statistical differences were observed in hospitalization rates or emergence times among the groups. These findings imply that the capability to evade immune responses emerges as the most pivotal antigenic trait determining a variant’s ability to sustain reproduction and facilitate widespread transmission, with infectivity playing a secondary role. Furthermore, the hospitalization rate does not appear to be a driving force in virus evolution, nor does it exhibit any directional changes throughout this process. Additionally, the absence of statistical differences in the emergence times of the four variant groups suggests that their antigenic alterations did not occur in a temporal sequence.

### 3.3 Epidemiological distribution characteristics of an epidemic

**Figure 5:**
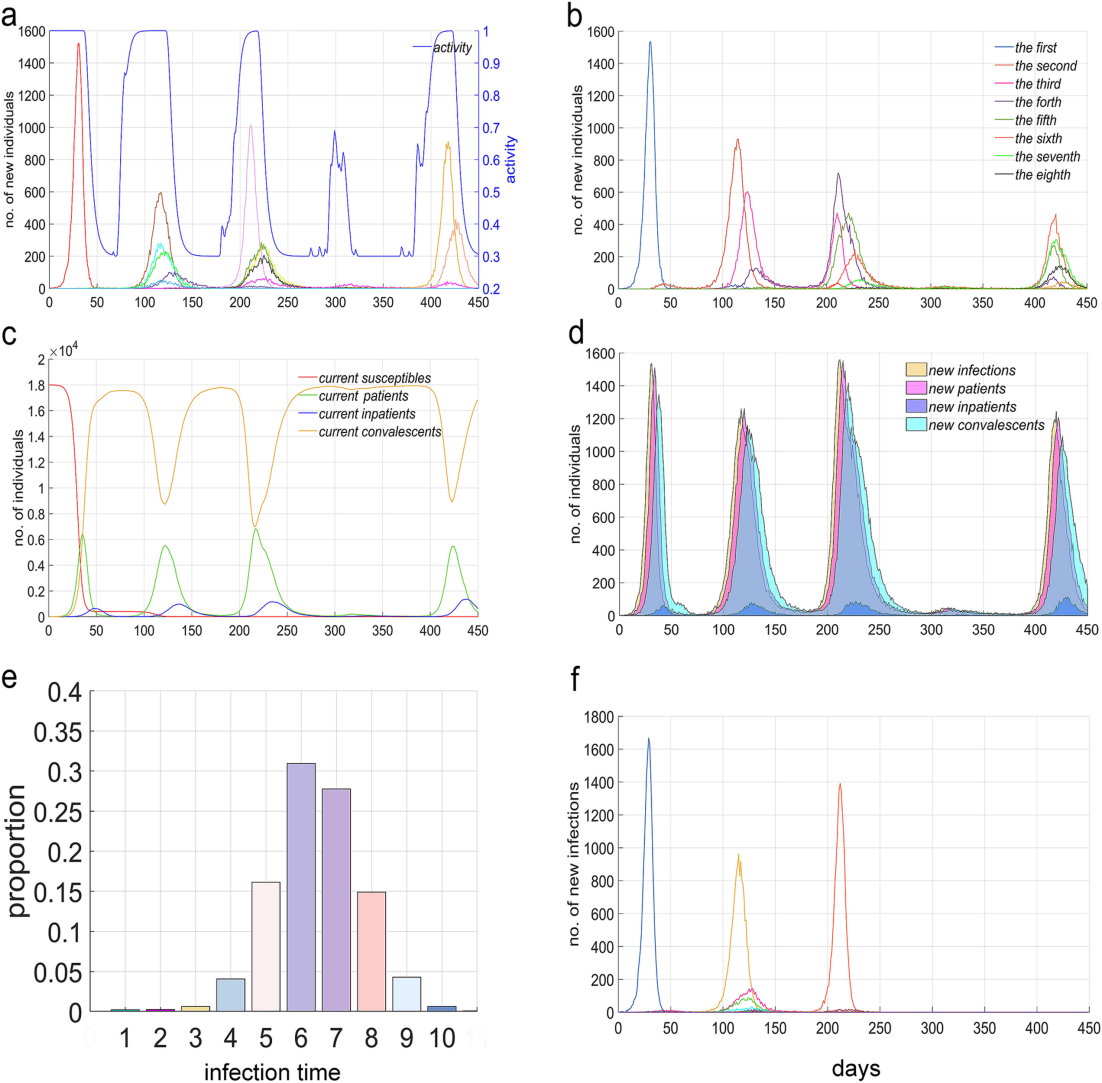
Epidemiological distribution characteristics of an epidemic from day 0 to day 450. **(a)** The temporal distribution of the number of new infections caused by different strains alongside the social activity level *β*(*t*). The number of infections is measured by the left vertical axis, while social activity level is measured by the right vertical axis. **(b)** The temporal distribution of new infections, categorized by the number of times infected. **(c)** The temporal distribution of the current number of susceptible individuals, unhospitalized patients (including mild cases and severe cases before hospitalization), hospitalized patients, and recovered individuals. **(d)** The temporal distribution of new infections, unhospitalized patients, hospitalized patients, and recovered individuals. **(e)** The composition ratio of infections, differentiated by the times of infections. **(a–e)** are all based on the premise that *β*_min_ = 0.3. **(f)** At *β*_min_ = 0.2, the temporal distribution of the number of new infections caused by different strains.

Figure 5a illustrates the temporal pattern of the number of infections attributable to major variant strains, juxtaposed with the trend in social activity levels. Upon examination of this figure, four distinct periodic outbreaks of the epidemic are evident. The first outbreak was characterized by the dominance of a single prevalent strain; however, subsequent outbreaks exhibited a more diverse array of prevalent strains, comprising a dominant strain along with several minor ones. Certain strains rapidly ascended to dominance upon their emergence, whereas others required a prolonged period of low-level transmission before becoming predominant. Concurrently, the temporal distribution of social activity levels mirrored the periodic fluctuations observed in the strains. It was found that outbreaks led to a decline in social activity, which subsequently returned to normal levels following the containment of the outbreak, only to be followed by a resurgence of the epidemic that once again reduced social activity.

Figure 5b presents the temporal distribution of infection counts, revealing four epidemic cycles that are analogous to those observed in Figure 5a. The majority of the infections exhibited at least two distinct epidemic cycles, whereas some, such as the first, second, and eighth infections, demonstrated only one significant outbreak period. Conversely, others, like the fourth infection, experienced three outbreak periods. This figure notably indicates that later epidemic cycles have accumulated a higher number of infections.

Figure 5c illustrates the current temporal distribution of various population groups. It depicts a rapid decline in the number of susceptible individuals following the first major outbreak, along with pronounced periodic variations in the counts of recovered individuals and those in the infectious period. Notably, these variations exhibit inverse trends.

Figure 5d demonstrates that the newly determined population sizes of the four groups exhibit consistent periodic fluctuations, with variations in peak times attributable to delays in infection, onset of symptoms, hospitalization, and recovery.

Figure 5e presents the composition ratio of the number of infections by different infection counts. It’s observed that infections occurring less than 4 times or more than 9 times constitute a smaller proportion, while those infected 5 to 8 times account for a higher percentage, reaching 90%. The results shown in Figures 5a–e were obtained at the lowest social activity level, *β_min_* = 0.3.

Figure 5f displays the temporal distribution of the number of infections caused by each strain when *β_min_* = 0.2, showing that a lower *β_min_* and stricter NPIs correspondingly reduce the number of variant strains, the cumulative number of infections, and the duration of the epidemic.

### 3.4 The impact of strain mutation rate and minimum social activity level on epidemic spread

**Figure 6:**
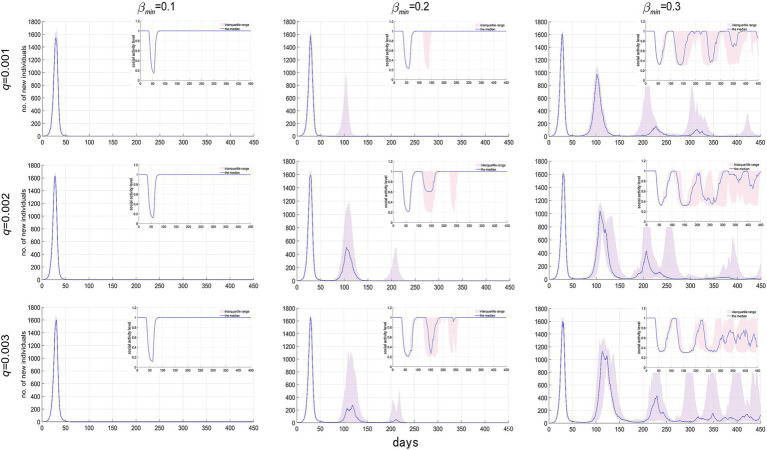
Temporal distribution of new infections and social activity level’s median and interquartile range with varying virus mutation rates and minimum social activity levels.

**Figure 7:**
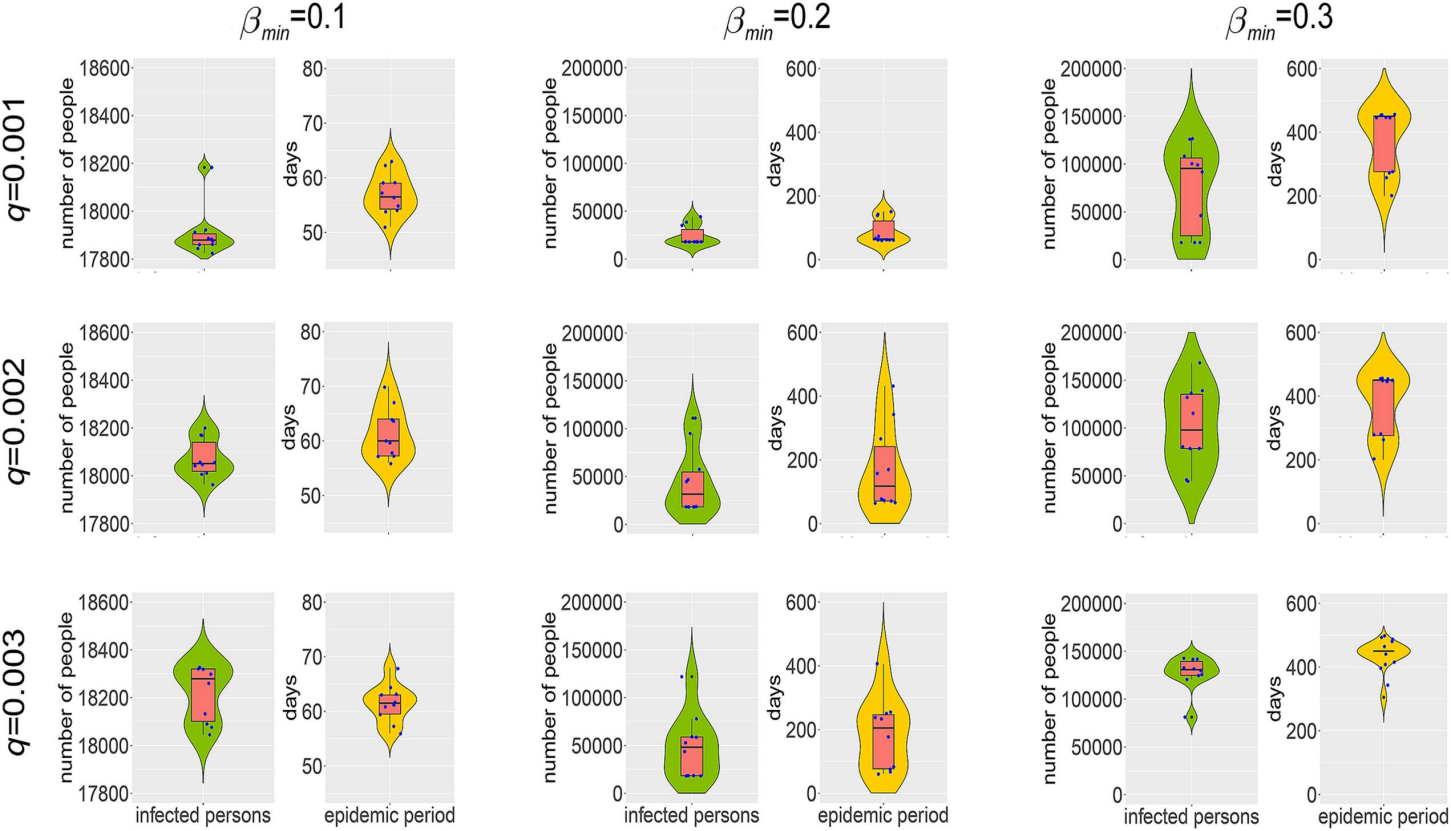
Temporal distribution of cumulative infections and epidemic duration’s median and interquartile range with varying virus mutation rates and minimum social activity levels.

The strain mutation rate (*q*) and the minimum social activity level *β_min_*, which measures the strictness of NPIs, represent the natural and societal factors affecting virus evolution and epidemic spread, respectively. Their impacts on the scale of the epidemic are illustrated in Figures 6, 7. From every row in Figures 6, 7, it’s clear that, holding *q* constant, a smaller *β_min_*, meaning stricter NPIs, significantly reduces the number of infections, the number of epidemic cycles, and the duration of the epidemic. Observing each column, at *β_min_* = 0.1, *q* has no significant impact on epidemic spread, indicating that the epidemic concludes after just one cycle; however, at *β_min_* = 0.2 and 0.3, an increase in *q* slightly raises the number of infections and extends the epidemic duration. These results demonstrate that compared to the virus mutation rate, the strictness of NPIs is a more crucial factor in determining the scale of an epidemic.

### 3.5 Sensitivity analysis

**Figure 8:**
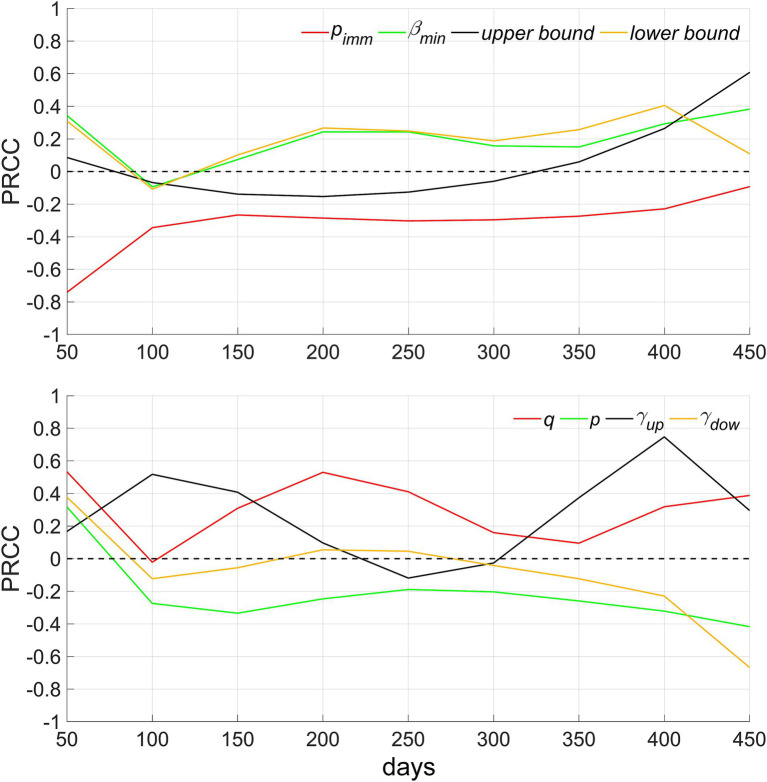
Sensitivity analysis of eight model parameters over continuous time. *p_imm_* denotes the proportion of the population possessing innate immunity, *β_min_* denotes the lowest point of decrease in social activity, upper and lower bound denote *ω_up_* and *ω_dow_*, respectively, *q* denotes the rate of strain mutation, *p* denotes genetic variability, *γ_up_* and *γ_dow_* denote the rate of recovery and decline in social activity, respectively.

In this investigation, sensitivity analyses were conducted on the model, utilizing eight main parameters along with a continuous time series for the total number of infected individuals. We sampled *N* = 50 instances from a uniform distribution within each parameter’s plausible range. The analysis employed Partial Rank Correlation Coefficients (PRCCs) for these parameters, which vary between-1 and 1. PRCC values approaching 1 or-1 signify a strong positive or negative influence of the parameter on the output, respectively. Conversely, a PRCC value nearing 0 suggests a minimal impact of the parameter on the model’s output (refer to Figure 8).

## 4 Discussion

### 4.1 Comparison of study conclusions with prior empirical research

Several virological and immunological studies have reported moderate escape of SARS-CoV-2 Alpha, Beta, Gamma, and Delta variants from vaccine-derived antibodies and convalescent sera in lab experiments (23–26). However, the Omicron ‘complex,’ including sublineages BA.1 to BA.5, can infect both vaccinated and previously infected individuals (27). Research indicates that recurrent Omicron outbreaks suggest antibody evasion is becoming a primary evolutionary trait, surpassing enhanced infectivity (27). Additionally, the *R0*, an infectivity indicator, has increased from 2.6 for the wildtype to 4.2 for Alpha, 7.2 for Delta, and 8.4 for Omicron (6), indicating increased transmissibility of SARS-CoV-2 VOCs, which exhibit enhanced immune evasion and infectivity.

Moreover, epidemiological studies have confirmed that the infection fatality ratios are 1.2, 3.0, 4.71, 0.4, 2.1, and 0.7% for the wildtype, Alpha, Beta, Gamma, Delta, and Omicron variants, respectively (6, 28, 29), reflecting considerable variability in their virulence without exhibiting a clear trend toward increased virulence. These scientific findings are congruent with the research conducted using our model, reinforcing the validity of our theoretical deductions through empirical evidence. This alignment underscores the scientific rigor and reliability of our research methodology and outcomes.

### 4.2 The role of other factors in NPIs

In addition to the hospitalization rate of infected individuals, indicators for assessing viral virulence also encompass the probability of death given infection and the probability of death given hospitalization for severe disease. To streamline analysis and exposition, this study posits that the hospitalization rate serves as the sole determinant for timing the implementation of NPIs. This simplifying assumption may introduce certain deviations between the model’s hypothetical scenarios and real – world circumstances.

Furthermore, apart from virulence, the virus transmissibility emerges as a pivotal factor influencing governmental interventions and individual protective behaviors. For instance, despite the Omicron variant exhibiting significantly reduced pathogenicity compared to its predecessors, its transmissibility has notably increased. Consequently, several countries adopt a cautious stance toward relaxing epidemic control measures, apprehensive that a surge in patient numbers could impose an enormous strain on the healthcare system. Moreover, since 2023, there has been a marked uptick in the incidence of respiratory infectious diseases in China (30). In response, the Chinese Center for Disease Control and Prevention has formulated an influenza vaccination strategy for the 2023–2024 influenza season (30). Additionally, the elevated incidence rates have spurred individuals to adopt more proactive self – protection measures and moderately increase social distancing (31). These studies underscore that the virus’s transmission speed can also influence social distancing practices, thereby impacting the trajectory of viral evolution. Whether the quantification of virus transmission speed exerts a comparable influence on the direction of viral evolution as the hospitalization rate remains a subject that warrants further research and empirical validation.

### 4.3 The role of NPIs in virus evolution direction

The evolutionary trajectory of SARS-CoV-2 has garnered sustained interest in both academic and public domains in recent years. Understanding this trajectory is not only pivotal for predicting epidemic trends and formulating public health strategies, but also for establishing a theoretical foundation for the scientific management of emerging infectious diseases in the future. The evolution of the virus is influenced by a complex interaction of both natural and societal factors. In this study, we consider the virus mutation rate as a representative natural factor and NPIs as societal factors. A genetic algorithm was used to simulate the virus’s evolutionary process. The findings reveal that NPIs are the primary determinant influencing the direction of viral evolution, with the mutation rate playing a secondary yet significant role.

An elevation in the mutation rate gives rise to a greater diversity of variants. Subsequently, NPIs selectively favor strains with enhanced immune evasion and infectivity, markedly increasing the probability of widespread infections within the population. Furthermore, the stringency of NPIs critically influences both the evolutionary path of the virus and the progression of the epidemic. Strict NPIs reduce human interactions, thereby impeding the virus’s capacity to spread. Conversely, more lenient NPIs lead to cyclical outbreaks due to the emergence of highly transmissible strains, resulting in a recurrent pattern of epidemic resurgence and the subsequent reimplementation of control measures.

### 4.4 The role of genetic distance in quantifying viral variation

As this research does not base itself on molecular biology, the analysis of the direction of virus evolution and the mechanisms of immune evasion is not approached from the perspectives of genomics and protein structures, or immunology. The ability of a variant to infect, replicate within, and disseminate among hosts is influenced by its genetic distance from previously circulating variants, rendering the evolution of antigenic novelty a pivotal factor in determining a variant’s reproductive success and fitness (27). Based on this principle, we have devised a function that quantifies the probability of reinfection as a function of genetic distance, to assess whether variants can cause reinfection in individuals who have recovered.

In this context, genetic distance pertains to the count of mutations in the genome sequences encoding the spike protein between two strains. As the genetic distance between two strains increases, so does the number of their genomic mutations, leading to a more phylogenetically distant relationship. Consequently, the likelihood that a variant can evade antibodies from previous infections and reinfect the host escalates. The computation of genetic distance is grounded in the structure of the phylogenetic tree. Given that each mutation at a genetic locus signifies the emergence of a new variant, resulting in an additional branch on the phylogenetic tree, the genetic distance can be ascertained by tallying the number of branches separating two strains within the phylogenetic tree (refer to Figure 3a). From this, the probability of reinfection can be determined.

### 4.5 The role of social distance in the spread of epidemics

In our analysis of the impact of NPIs on the epidemic, we categorized various measures as enhancements to social distancing, based on governmental practices in epidemic prevention and control. Specifically, the decision to implement NPIs was predicated on the hospitalization rate of new infections. While the enforcement of NPIs can indeed reduce social activities to some extent, achieving a complete absence of interpersonal contact is exceedingly challenging due to the considerable social management costs and economic repercussions involved. This reality imposes a limit on the degree to which social distancing can be increased, namely, the reduction of social activity to its minimum feasible level. This limit is indicative of the stringency of NPIs implementation, which exhibits marked variations across different countries and regions.

For instance, following the outbreak of COVID-19 in Wuhan in December 2019, the Chinese government enforced a series of stringent NPIs that markedly curtailed people’s movements, thereby reducing the effective reproduction number to nearly zero (32, 33). These measures remained in place until April of the subsequent year, when the outbreak was effectively contained without any significant rebound for an extended period. In contrast, some countries opted for a more moderate approach, intensifying (or easing) containment measures in response to rising (or falling) hospitalization rates. Meanwhile, some developing countries struggled to promptly implement effective epidemic prevention measures owing to inadequate testing capabilities. Given that the majority of countries adopted moderate epidemic control measures, we incorporated this approach into our model and devised a social activity function to mirror changes in social distancing. Specifically, individuals increase their social distancing (and decrease their social activity) when the hospitalization rate exceeds an upper threshold, and they decrease their social distancing (and increase their social activity) when the hospitalization rate falls below a lower threshold.

### 4.6 Relationship between intervention strategies and epidemic trends

The research reveals that the cyclical outbreaks of the epidemic are instigated by the persistent emergence of variants with heightened transmissibility, coupled with the limited scope of changes in social distancing behaviors. When social distancing is increased, the resistance to virus transmission intensifies, leading to a reduction in the speed of epidemic spread and maintaining a low level of prevalence. However, upon relaxation of these measures due to improvements in the epidemic situation, the variants rapidly accelerate their spread, thereby initiating a new outbreak cycle. The findings indicate that in the event of a novel infectious disease emergence, the implementation of stringent epidemic prevention measures in the short term enhances the probability of complete disease eradication, albeit at the expense of sacrificing immediate economic objectives and incurring elevated social management costs. Conversely, opting for moderate prevention measures may entail bearing the ongoing social costs and health risks associated with the cyclical recurrence of epidemic outbreaks.

### 4.7 Limitations of the study

Firstly, computational constraints limited our model’s population size, as individual-based models require more memory than differential equation models. Secondly, NPIs were implemented based only on hospitalization rates, neglecting other indicators, and their effects were uniformly attributed to increased social distancing, potentially reducing model accuracy. Thirdly, functions for estimating reinfection rates and social activity fluctuations were not calibrated with survey data, requiring further validation. Fourthly, the model’s mutation rate was adjusted beyond real-world observations to highlight outbreak cyclicity (34). Fifthly, the model is a theoretical exploration of virus evolution under human intervention, not grounded in actual COVID-19 data, potentially impacting its realism. Lastly, virulence evolution’s predictability is complex, influenced by factors like within-host competition, vaccination, and immune interactions (35), which were not considered in this study.

In summary, using SARS-CoV-2 as a case study, our research employs computer simulations to present a holistic view of epidemic dissemination, viral mutation, and the enforcement of control measures. It explores the underlying logic governing the interplay between viral mutations and NPIs, analyzes the trajectories of viral evolution, and provides actionable recommendations for prevention and control strategies. Our findings reveal that strains capable of widespread transmission demonstrate markedly enhanced immune evasion capabilities and elevated infectivity relative to other strains. This antigenic evolution transpires under the selective pressure exerted by NPIs; however, the virulence and emergence timing of these strains exhibit no discernible correlation with NPIs. In the event of an outbreak, the stricter the implementation of NPIs, the more effectively the epidemic’s spread and viral mutation can be contained; conversely, lax measures may give rise to cyclical outbreaks owing to the continuous emergence of new variants. Although the reduced virulence of the Omicron variant and increased population immunity have significantly diminished the threat posed by SARS-CoV-2, it remains imperative to continuously monitor antigenic changes in variants to preempt the emergence and outbreak of variants with heightened virulence and transmissibility. The conclusions derived from this research hold relevance not only for SARS-CoV-2 but also for influenza and potential future pathogens characterized by high mutation rates.

## Data Availability

The datasets presented in this study can be found in online repositories. The names of the repository/repositories and accession number(s) can be found in the article/Supplementary material.

## Supplementary material

APPENDIX I The framework of quantitatively analyzing the relationship between non-pharmaceutical interventions and the direction of virus evolution using a dynamic model. (DOI:10.17632/r7ft6ggy78.1).

APPENDIX II The sensitivity analysis of quantitatively analyzing the relationship between non-pharmaceutical interventions and the direction of virus evolution using a dynamic model. (DOI:10.17632/g67n3x7nj7.1).
