# Supplementary material for: Quantitatively analyzing the relationship between non-pharmaceutical interventions and the direction of virus evolution using a dynamic model
Source: Front Public Health. 2025 May 9;13:1542759. doi: 10.3389/fpubh.2025.1542759 (PMC12098584; doi:10.3389/fpubh.2025.1542759)
Supplement: APPENDIX I — The framework of quantitatively analyzing the relationship between non-pharmaceutical interventions and the direction of virus evolution using a dynamic model. (DOI:10.17632/r7ft6ggy78.1). [file Data_Sheet_1.pdf]

```
%clear all
np=20000;                                  %Total population
proportion=[0.276,0.284,0.292,0.148];      % The proportion of age groups: 0-24, 25-44, 45-64, 65+
chr=[0.0033,0.0145,0.0331,0.0789];         % Severity rate by age group
na=np*proportion;                          % Population by age group
R0=[2,5];                                  % The range of values for the basic reproduction number
lambda=[0.6,1.6];                          % The range of values for immune escape index lambda
hr=[0.5,5];                                % The coefficient representing hospitalization rates
p_imm=0.1;                                 % The proportion of people with congenital immunity
mu_inc=3.1;                                % Average incubation period
sd_inc=2.6;                                % Standard deviation of incubation period
q=0.003;                                   % Probability of strain mutation
p=0.1;                                     % Isolation of strain mutations
delta=0.0467;                              % Mortality rate of inpatients
gamma_dow=0.2;                             % R0 decline rate, gamma low negatively correlated with R0
gamma_up=0.3;                              % R0 rise rate, gamma up is positively correlated with R0
p_bot=0.1;                                 % The percentage of R0 decreasing to its maximum value
thr_hos=0.05;                              % Hospitalization rate warning value
unthr_hos=0.04;                            % Hospitalization rate release warning value
name=1000;                                 % Gene sequence cardinality
cycle=10;                                  % The number of large loops
n0=1;                                      % Initial number of strains
daymax=450;                                % Duration of disease transmission

% Start a big loop
New_inf=[];
Par=[];
N_tree=[];
[rN,cN]=size(New_inf);

while rN<10
    hos_Inf=[];                                % Store hospitalization rates for newly infected individuals
```

```
% Establish a population file Fp, with each row representing one person. Each column includes:
% 1 age, 2 congenital immunity, 3 number of previous infections, 4 type of strain
Fp=zeros(np,4);
% The first column is divided into four age groups
c1=[];
for i=1:length(na)
    c1=[c1;ones(round(na(i)),1)*i];
end
Fp(:,1)=c1;

% 10% of the second column has innate immunity, represented by 1
r1=randperm(np);
id_imm=r1(1:p_imm*np);
id_sus=1:np;
id_sus(id_imm)=[];
Fp(id_imm,2)=1;

par_T=[];
D=[];                                   % Store infected person information
% Initially, there were n0 strains, R0_1, lambda_1, hr_1, and they were stored in the Tree
name0=[name:name+n0-1];
R0_1=5;
lambda_1=median(lambda);
hr_1=median(hr);                        % The coefficient of the severity rate of this strain
Tree=[];                                % Each line stores: names, R0, lambda, hr, and generation time
Tree(:,1)=name0;Tree(:,2)=R0_1;Tree(:,3)=lambda_1;Tree(:,4)=hr_1;Tree(:,5)=zeros(n0,1);
E=[];                                   % Store the source of infection (line 1) and the time of infection (line 2)

for j=1:n0
    %First case id_1
    r_sus=randperm(length(id_sus));
    id_1=id_sus(r_sus(1));
```

```
    id_sus(r_sus(1))=[];                    % Remove the first case from susceptible individuals
  % 1 infected person, 2 infectious sources, 3 strains, 4 infection frequency, 5 age, 6 hospitalization (0 not hospitalized, 1 hospitalized, 2 deaths), 7 infection time, 8 incubation period,
  % 9 infection period, 10 admission time, 11 discharge time (end of infection period, time of death), 12 current infection status (1 infection, 2 recovery, 3 death), 13 whether it has been
  % transmitted to others (0 no, 1 yes)
    D(j,1)=id_1;D(j,2)=0;D(j,3)=name+j-1;D(j,4)=1;D(j,5)=Fp(id_1,1);
    D(j,7)=0;D(j,8)=0;D(j,12)=1;D(j,13)=0;
    % Determine whether to be hospitalized
    age=Fp(id_1,1);
    hr_2=chr(age)*hr_1;                     % Judging hospitalization rate based on age
    fh=binornd(1,hr_2);                     % Determine whether to be hospitalized
    % Infection period and length of hospital stay
    if fh==1                                % If hospitalized
        per_inf=rand*(12-4)+4;              % From onset to hospitalization time
        per_hos=rand*(20-12)+12;            % Hospitalization period
        % Judging whether there is death
        dr=binornd(1,delta);
        if dr==1                            % If death occurs
            D(j,6)=2;
        else
            D(j,6)=1;
        end
        D(j,9)=per_inf;                     % infection period
        D(j,10)=D(j,9);                     % hospital stay
        D(j,11)=D(j,10)+per_hos;            % Discharge time
    else
        D(j,6)=0;
        D(j,9)=rand*(7-2)+2;                % Infectious period without hospitalization
        D(j,10)=0;
        D(j,11)=D(j,9);
    end

    % Update the Fp of the first case, 3 previous infections, 4 strain types
```

```
        Fp(id_1,3)=1;
        Fp(id_1,4)=D(j,3);
        nu_inf=poissrnd(Tree(j,2));               % The number of effective contacts of the first case during the infectious period
        D(j,13)=1;

        if nu_inf>0
            times_inf=rand(1,nu_inf)*D(j,9);       % Next generation infection time point
            E0=[ones(1,nu_inf)*D(j,1);times_inf];  % Store the information of the first generation of infected individuals in
                                                   % E, with the first line indicating the source of infection and the second
                                                   % line indicating the time of infection
            E=[E,E0];
        end
    end

    [rE,cE]=size(E);
    if cE>0
        t=min(E(2,:));
        index_t=find(E(2,:)==t);                    % The serial number where t is located
        e=E(:,index_t);                             % Use e to represent this column
        E(:,index_t)=[];                            % Remove this column
        t1=0;                                       % Store the time for issuing alerts
        t2=0;                                       % Store the time to release the alert
        Par_t=[];                                   % Store par-R0 (line 1) and t (line 2) at time t
        Par_t(:,1)=[1;0];                           % The initial value is [1; 0], par-R0 in line 1, t in line 2
        tt=1;                                       % The number of days the storage has been running until
        New_Tre=[];                                 % Store the number of newly infected individuals for each strain, with each
                                                    % row representing one strain
       id_rec=[];                                  % Storing rehabilitation patients
    end

    % Start small loop
    while cE>0 & t<=daymax
```

```
% Update the infection status in D first
f1=find(D(:,11)<t & D(:,12)==1 & D(:,6)~=2); % Identify individuals in D who have recovered but are still infected
D(f1,12)=2;                                  % Change their status to rehabilitation
id_rec=[id_rec,D(f1,1)’];                    % Store rehabilitation patient ID
f2=find(D(:,11)<=t & D(:,12)==1 & D(:,6)==2);% Find individuals in D who have died but are still in an infected state
D(f2,12)=3;                                  % Change their status to death
id_inf=D(find(D(:,12)==1),1)’;               % Being in the stage of infection
id_hos=D(find(D(:,6)~=0 & D(:,10)<t & t<=D(:,11)),1)’; % Hospitalized

% Selecting the next generation of infected individuals can only be done from id_sus, id-rec, id-inf, and id_imm
id_cum=[id_sus,id_rec,id_inf,id_imm];
r_next=round(rand*length(id_cum)+0.5);
% Determine whether transmission will occur
id_sr=[id_sus,id_rec];
f_s=find(D(:,1)==e(1) & D(:,12)==1);         % Find e (1) who is currently in the infectious phase
strain0=D(f_s,3);                            % E (1) is the ID of the currently infected strain

if r_next<=length(id_sr)                     % If there is a possibility of transmission
    % Determine whether the strain has mutated
    r_var=binornd(1,q);
    fT=find(Tree(:,1)==strain0);              % The row where the strain is located in the Tree

    if r_var==1                               % If mutated
        % Determine the name of the new strain
        if mod(max(Tree(:,1)),10)==0
            b=ceil(log10(max(Tree(:,1))))+1;   % The maximum number of digits in the Tree
        else
            b=ceil(log10(max(Tree(:,1))));     % The maximum number of digits in the Tree
        end
        Tree0=num2str(Tree(:,1),b);            % Convert Types to Strings
        Tree0(find(Tree0==’ ’))=’0’;
        s_T=size(Tree0);
```

```
if mod(strain0,10)==0
    b0=ceil(log10(strain0))+1;
else
    b0=ceil(log10(strain0));
end
strain0=num2str(strain0,b0);
if str2num(strain0)==name
    f_1000=find(Tree(:,1)<name*2);
    strain_1=max(Tree(f_1000,1))+1;
else
    family=[];
    k=1;
    while k<=s_T(1)                  % Find the strin0 family for each row of Tree0
        f0=min(find(Tree0(k,:)>’0’));
        f1=f0-1+length(strain0);
        if f1<=s_T(2) & str2num(Tree0(k,f0:f1))==str2num(strain0)
            family=[family;str2num(Tree0(k,f0:s_T(2)))];
        end
        k=k+1;
    end
    f2=find(str2num(strain0)<=family & family<=str2num(strain0)*name+(name-1));
    family1=family(f2);

    if length(family1)==1
        strain_1=str2num(strain0)*name+1;
    else
        strain_1=max(family1)+1;
    end
end

% Determine the new R0, lambda, and hr, and the mutation strategy is to mutate all three indicators, with a
 % variation amplitude exceeding the original p-fold
```

```
    Ranges=[R0;lambda;hr];
    pres=Tree(fT,2:4);                                 % Previous variable values
    news=[];                                           % Store new variables
    for j=1:3                                          % Select new values for each variable individually
        val_new=rand*(Ranges(j,2)-Ranges(j,1))+Ranges(j,1);   % New variable values
        val_pre=pres(j);
        while abs(val_new-val_pre)/val_pre<p           % If the mutation distance of the strain is less than p
            val_new=rand*(Ranges(j,2)-Ranges(j,1))+Ranges(j,1);% New variable values
        end
        news(j)=val_new;
    end
    % Store the information of the new strain in a tree
    tree=[];
    tree(1:4)=[news,t];
    tree=[strain_1,tree];
    Tree=[Tree;tree];                    % Merge Tree with Tree
    R0_1=tree(2);
    lambda_1=tree(3);
    hr_1=tree(4);
else                                     % If there is no mutation
    strain_1=strain0;
    R0_1=Tree(fT,2);
    lambda_1=Tree(fT,3);
    hr_1=Tree(fT,4);
end

% Determine the type of person in contact (sus or rec)
distance=[];                  % Store the genetic distance between strin_1 and other strains of the virus
if r_next>length(id_sus)      % If the person in contact is a rehabilitation patient
    % Determine whether the rehabilitation patient will be reinfected
    % Find the infected strain of the recovered individual
    state0=2;                 % Indicates that the contact person is a rehabilitation patient
```

```
id_infected=id_sr(r_next);             % Rehabilitation patient ID
f_r=find(D(:,1)==id_infected & D(:,12)==2); % The rehabilitation of patients in D
type=D(f_r,3);                         % Types of strains previously infected by recovered individuals
type=unique(type);                     % Remove identical elements
Types0=[strain_1;type];                % Merge the infectious strain with the recovered strain
% Calculate the genetic distance between the infectious source strain and the infected person strain
% Disassemble the strains of Types and store the gene loci of each strain in each column of Strains
if mod(max(Types0),10)==0
    b=ceil(log10(max(Types0)))+1;      % The maximum number of digits in Types
else
    b=ceil(log10(max(Types0)));        % The maximum number of digits in Types
end
Types=num2str(Types0,b);               % Convert Types to Strings
Types(find(Types==’ ’))=’0’;
Strains=[];                            % Store the split gene sequence and store each gene fragment in each column
 size_T=size(Types);
for k=1:size_T(1)                      % Split each row of Types
    k0=1;
    c_min=min(find(Types(k,:)>’0’));
    Strains(k,k0)=str2num(Types(k,c_min));
    for k1=1:(size_T(2)-c_min)/3
        k0=k0+1;
        Strains(k,k0)=str2num(Types(k,c_min+[1:3]+3*(k1-1)));
    end
end

for k=2:size_T(1)
    if Strains(1,:)==Strains(k,:)
        distance0=0;
    else
        k0=1;                                  % the k0th gene locus
        while Strains(1,k0)==Strains(k,k0)
```

```
                k0=k0+1;
            end
            if Strains(1,k0)==0 | Strains(k,k0)==0  % If one party's genes show 0
                % The difference in gene length between the two is the distance
                distance0=abs(max(find(Strains(1,:)>0))-max(find(Strains(k,:)>0)));
            else
                % If the genes of the two appear different on k0-1
                d1=max(find(Strains(1,:)>0))-(k0-1);
                d2=max(find(Strains(k,:)>0))-(k0-1);
                distance0=d1+d2;
            end
        end
        distance(k-1)=distance0;
    end
else                                 % If the person in contact is a susceptible individual
    state0=1;                        % Indicates that the contact person is susceptible
    id_infected=id_sr(r_next);
    distance=1000;                   % If the patient is susceptible, the distance is 1000, which means the infection rate is 1
end

% Calculate the probability of infection under distance
p_inf=1-exp(-lambda_1*distance); % Probability of being infected under distance
p0=prod(p_inf);                  % P0 represents the probability of the contact being infected
inf0=binornd(1,p0);              % Indicates whether infected or not
if inf0==1                       % If an infection occurs
    d=[];
    d(1)=id_infected;
    d(2)=e(1);
    d(3)=strain_1;
    % Determine the number of infections
    d(4)=length(find(D(:,1)==d(1)))+1;
    % Update Fp, id_sus, id_rec
```

```
Fp(d(1),3)=d(4);                    % Update infection count
Fp(d(1),3+d(4))=d(3);               % Add new strains of the virus
if state0==1                        % Infected individuals are susceptible individuals
    id_sus(find(id_sus==d(1)))=[];
else
    id_rec(find(id_rec==d(1)))=[];
end
d(5)=Fp(d(1),1);                    % age
d(7)=e(2);
%潜伏期
inc_per=normrnd(mu_inc,sd_inc);     % Incubation period mu_inc=3.1;sd_inc=2.6;
while inc_per<1 | inc_per>5         % Keep the incubation period between 1-5 days
    inc_per=normrnd(mu_inc,sd_inc);
end
d(8)=inc_per;
% Whether hospitalized or deceased
hr_2=chr(d(5))*hr_1;                % Judging hospitalization rate based on age
fh=binornd(1,hr_2);                 % Determine whether to be hospitalized
% Infection period and length of hospital stay
if fh==1                            % If hospitalized
    % Judging whether there is death
    dr=binornd(1,delta);
    if dr==1                        % If death occurs
        d(6)=2;
    else
        d(6)=1;
    end
    d(9)=rand*(12-4)+4;             % From onset to hospitalization time
    d(10)=d(7)+d(8)+d(9);           % hospital stay
    per_hos=rand*(20-12)+12;        % Hospitalization period
    d(11)=d(10)+per_hos;            % Discharge time
else                                % If not hospitalized
```

```
            d(6)=0;
            d(9)=rand*(7-2)+2;              % Infectious period without hospitalization
            d(10)=0;
            d(11)=d(7)+d(8)+d(9);           % rehabilitation time
        end
        d(12)=1;                            % The current status is infected
        d(13)=0;
        D=[D;d];
    end
end

% Determine the number of effective contacts of all infectious sources at time t during the infection period
% First, determine the coefficient par-R0 of R0 at time t
% Determine the hospitalization rate of newly infected individuals at time t. If the hospitalization rate exceeds
% Thr_hos, an alert will be issued
day=floor(t);
new_hos=length(find(D(:,6)~=0 & day-1<=D(:,10) & D(:,10)<day));  % Newly added inpatients
new_inf=length(find(day-1<=D(:,7) & D(:,7)<day));                % Calculate the new hospitalization rate
% Calculate the new hospitalization rate
if new_inf>0
    hos_Inf=[hos_Inf,new_hos/new_inf];
else
    hos_Inf=[hos_Inf,0];
end
r_min=max(length(hos_Inf)-4,1);
hos_inf=mean(hos_Inf(r_min:length(hos_Inf)));

if t1==0 & t2==0 & hos_inf>=thr_hos             % Issue a warning
    t1=t;
elseif t1==0 & t2==0 & hos_inf<thr_hos          % If no warning is issued
    par_t=[1;t];
    par_max=1;
```

```
        end
        if t1>0 & t2==0 & hos_inf>=unthr_hos
            % If a warning has been issued and the hospitalization rate is greater than the warning release value
            t_=t-t1;
            par_R0=(par_max-p_bot)*2./(exp(-gamma_dow*t_)+exp(gamma_dow*t_))+p_bot;
            par_t=[par_R0;t];
        elseif t1>0 & t2==0 & hos_inf<unthr_hos
            % If a warning has been issued and the hospitalization rate reaches the warning lifting value
            t_=t-t1;
            par_min=(par_max-p_bot)*2./(exp(-gamma_dow*t_)+exp(gamma_dow*t_))+p_bot;
            par_t=[par_min;t];
            t1=0;                                   % Clear the previous t1
            t2=t;
        elseif t2>0 & t1==0 & hos_inf<thr_hos
            % If the warning is lifted and the hospitalization rate is less than the warning value
            t_=t-t2;
            par_R0=1-2*(1-par_min)/(exp(-gamma_up*t_)+exp(gamma_up*t_));
            par_t=[par_R0;t];
        elseif t2>0 & t1==0 & hos_inf>=thr_hos
            % If the number of inpatients reaches the warning value again after the warning is lifted
            t_=t-t2;
            par_max=1-2*(1-par_min)/(exp(-gamma_up*t_)+exp(gamma_up*t_));
            par_t=[par_max;t];
            t2=0;
            t1=t;
        end
        Par_t=[Par_t,par_t];

        % Calculate the transmission time of all infectious sources one by one at time t
        % At time t, the row where the source of infection is located, r-inf
        r_inf=find(D(:,7)+D(:,8)<=t & t<=D(:,7)+D(:,8)+D(:,9) & D(:,13)==0);
        D(r_inf,13)=1;                      % Update the status of infection
```

```
id_infectors=D(r_inf,1);            % Infectious source ids
for j=1:length(id_infectors)        % Determine the next generation's infection time based on infection sources
    % Find the strain var0 and its R0_var0 of the infectious source
    var0=D(r_inf(j),3);
    R0_var0=Tree(find(Tree(:,1)==var0),2);
    % Calculate parRt at time t
    % The population that may be infected by the source of infection at time t includes: susceptible individuals,
    % immunized individuals, infected individuals, recovered individuals, and hospitalized individuals (excluding)
    id_dea=D(find(D(:,12)==3),1);
    p_1=(length(id_hos)+length(id_dea))/np;
    p_Rt=Par_t(1,find(Par_t(2,:)==t));              % Par_20 at time t
    p_Rt=(1-p_1)*p_Rt;
    Rt=R0_var0*p_Rt;                                % Rt at time t
    nu_inf=poissrnd(Rt);                            % Number of effective contacts during the infectious period
    if nu_inf>0
        % Determine the time of infection, D: 7 infection time, 8 incubation period, 9 infection period
        time_inf=rand(1,nu_inf)*D(r_inf(j),9)+D(r_inf(j),7)+D(r_inf(j),8);
        Time_inf=[ones(1,nu_inf)*id_infectors(j);time_inf];
        E=[E,Time_inf];
    end
end
t=min(E(2,:));
index_t=find(E(2,:)==t);
e=E(:,index_t);
E(:,index_t)=[];
[rE,cE]=size(E);

% Display program progress by day
if fix(t)+1~=tt
    tt=fix(t)+1;
    tt
    sortrows(Tree(:,1))'
```

```
        end
    end

    new_inf=[];    % Store new infected individuals
    for j=1:ceil(max(D(:,7)))
        new_inf=[new_inf,length(find(j-1<=D(:,7) & D(:,7)<j))];
    end

    if sum(new_inf)>=100
        rN=rN+1;
        New_inf(rN,1:length(new_inf))=new_inf;
        %对 Par_t 进行简化
        for j=1:ceil(max(D(:,7)))
            f1=find(j-1<=Par_t(2,:) & Par_t(2,:)<j);
            m1=mean(Par_t(1,f1));
            Par(rN,j)=m1;
        end
        New_inf
        N_tree(rN)=length(Tree(:,1));
    end

    % Count the total number of people infected with each strain of virus
    for j=1:length(Tree(:,1))
        Tree(j,6)=length(find(D(:,3)==Tree(j,1)));
    end
end
xlswrite('C:\Users\LENOVO\Desktop\New_inf.xlsx',New_inf)
xlswrite('C:\Users\LENOVO\Desktop\Par.xlsx',Par)


% Draw the time distribution of newly infected individuals of each strain
New_Tre=xlsread('C:\Users\LENOVO\Desktop\New_Tre1.xlsx');
```

```
D=xlsread(’C:\Users\LENOVO\Desktop\D1.xlsx’);
t0=1:ceil(max(D(:,7)));
hold on
New_Tre0=New_Tre;
New_Tre0(:,1)=[];
plot(t0,New_Tre0,'LineWidth',1)
daymax=450;
xmax=daymax;
ymax=1800;
xlabel(’days’,’fontname’,’arial’,’fontsize’,20);
ylabel(’no. of new infections’,’fontname’,’arial’,’fontsize’,20);
set (gca,’fontsize’,20,’position’,[0.13,0.17,0.71,0.8] );
% Represent left boundary, bottom boundary, width, and height respectively
xlim([0 xmax])
set(gca,’Xtick’,[])
set(gca,’Xtick’,[0:xmax/9:xmax]);
ylim([0 ymax])
set(gca,’Ytick’,[0:ymax/9:ymax]);
box off
grid on

% Current susceptible, currently infected, currently hospitalized, currently recovering
% Susceptible person
D=xlsread(’C:\Users\LENOVO\Desktop\D1.xlsx’);
D0=D;
cur_sus=[];
for j=1:ceil(max(D(:,7)))
    f1=find(D0(:,7)<=j);
    num_t=18000-length(unique(D0(f1,1)));
    cur_sus=[cur_sus,num_t];
end
% Susceptibles
```

```
cur_inf=[];
for j=1:ceil(max(D(:,7)))
    f1=find(D(:,7)+D(:,8)<j & j<=D(:,7)+D(:,8)+D(:,9));
    cur_inf=[cur_inf,length(f1)];
end
% inmate
cur_hos=xlsread('C:\Users\LENOVO\Desktop\cur_hos1.xlsx');
% Rehabilitation patients
cur_rec=xlsread('C:\Users\LENOVO\Desktop\cur_rec1.xlsx');
cur_rec(length(cur_rec))=cur_rec(length(cur_rec)-1);

% draw designs
t0=1:ceil(max(D(:,7)));
hold on
p1=plot(t0,cur_sus,'r','LineWidth',1)
p2=plot(t0,cur_inf,'g','LineWidth',1)
p3=plot(t0,cur_hos,'b','LineWidth',1)
p4=plot(t0,cur_rec,'y','LineWidth',1)
legend('\fontsize{20}\itcurrent susceptibles','\fontsize{20}\itcurrent infections','\fontsize{20}\itcurrent inpatients','\fontsize{20}\itcurrent convalescents','NorthEast','box','off')
daymax=450;
xmax=daymax;
ymax=20000;
ylabel('no. of individuals','fontname','arial','fontsize',20);
set (gca,'fontsize',20,'position',[0.13,0.17,0.71,0.8] );
% Represent left boundary, bottom boundary, width, and height respectively
xlim([0 xmax])
set(gca,'Xtick',[])
set(gca,'Xtick',[0:xmax/9:xmax]);
ylim([0 ymax])
set(gca,'Ytick',[0:ymax/10:ymax]);
box off
grid on
```

```
% Draw new infected individuals, new patients, new inpatients, and new rehabilitation patients
D=xlsread('C:\Users\LENOVO\Desktop\D1.xlsx');
t0=1:ceil(max(D(:,7)));
t1=[t0,t0(end:-1:1)];
y=[sum(New_Tre0),zeros(1,length(t0))];
hold on
f1=fill(t1,y,'y','facealpha',0.4)   % New infected person, yellow
% Newly added patients
new_ill=xlsread('C:\Users\LENOVO\Desktop\new_ill1.xlsx');
y=[new_ill,zeros(1,length(t0))];
f2=fill(t1,y,'g','facealpha',0.4)   % Newly added patients, green
% Newly added inpatients
new_hos=xlsread('C:\Users\LENOVO\Desktop\new_hos1.xlsx');
y=[new_hos,zeros(1,length(t0))];
f3=fill(t1,y,'b','facealpha',0.4)   % New inpatients, blue
% Newly added rehabilitation patients
new_rec=[];
for j=1:ceil(max(D(:,7)))
    f1=find(j-1<D(:,11) & D(:,11)<=j);
    new_rec=[new_rec,length(f1)];
end
y=[new_rec,zeros(1,length(t0))];
f4=fill(t1,y,'b','facealpha',0.4)   % New inpatients, black

legend('\fontsize{20}\itnew infections','\fontsize{20}\itnew patients','\fontsize{20}\itnew inpatients','\fontsize{20}\itnew convalescents','NorthEast','box','off')
xmax=450;
ymax=1600;
ylabel('no. of individuals','fontname','arial','fontsize',20);
set (gca,'fontsize',20,'position',[0.13,0.17,0.71,0.8] );
xlim([0 xmax])
set(gca,'Xtick',[])
```

```
set(gca,'Xtick',[0:xmax/9:xmax]);
ylim([0 ymax])
set(gca,'Ytick',[0:ymax/8:ymax]);
box off
grid on

% Bar chart of infection frequency of infected individuals
D0=D;
max_inf=max(D0(:,4));
nre=zeros(1,max_inf);
while length(D0(:,1))>0
    f1=find(D0(:,1)==D0(1,1));
    n_inf=max(D0(f1,4));
    nre(n_inf)=nre(n_inf)+1;
    D0(f1,:)=[];
end
hold on
y=nre/sum(nre);
colors={'c','m','g','r','g','m','c','b','k','k'};
for j=1:max_inf
    bar([j],y(j),'FaceColor', colors{j});
end
xmax=11;
xlim([0 xmax])
set(gca,'Xtick',[])
set(gca,'Xtick',[0:xmax/11:xmax]);
ymax=0.4;
ylim([0 ymax])
set(gca,'Ytick',[])
set(gca,'Ytick',[0:ymax/8:ymax]);
xlabel('infection times','fontname','arial','fontsize',20);
ylabel('proportion','fontname','arial','fontsize',20);
```

```
set(gca,’fontsize’,20);
grid on
xlswrite(’C:\Users\LENOVO\Desktop\nre1.xlsx’,nre)

% Draw the time distribution of each infection
D=xlsread(’C:\Users\LENOVO\Desktop\D1.xlsx’);
D0=D;
N_rei=[];% Store the time distribution of newly infected individuals in waves 1 to n, with the jth row representing the jth infection
max_inf=max(D0(:,4));
daymax=450;
for j=1:max_inf
    fj=find(D0(:,4)==j);
    t_inf=D0(fj,7);
    for day=1:ceil(max(D(:,7)))
        N_rei(j,day)=length(find(day-1<=t_inf & t_inf<day));
    end
end
hold on
t0=1:ceil(max(D(:,7)));
plot(t0,N_rei,’LineWidth’,1)
legend(’the first’,’the second’,’the third’,’the forth’,’the fifth’,’the sixth’,’the seventh’,’the eighth’, ’FontSize’, 20,’box’,’off’)
xmax=daymax;
xlim([0 xmax])
set(gca,’Xtick’,[])
set(gca,’Xtick’,[0:xmax/9:xmax]);
ymax=1600;
ylim([0 ymax])
set(gca,’Ytick’,[])
set(gca,’Ytick’,[0:ymax/8:ymax]);
xlabel(’days’,’fontname’,’arial’,’fontsize’,20);
ylabel(’no. of new individuals’,’fontname’,’arial’,’fontsize’,20);
set(gca,’fontsize’,20);        % Represent left boundary, bottom boundary, width, and height respectively
```

```
grid on
xlswrite('C:\Users\LENOVO\Desktop\N_rei1.xlsx',N_rei)

% Draw the time distribution of Par_t
plot(Par_t(2,:),Par_t(1,:),'b','LineWidth',1)
xlim([1,ceil(max(D(:,7)))])
ylim([0,1])
xmax=daymax;
xlim([0 xmax])
set(gca,'Xtick',[])
set(gca,'Xtick',[0:xmax/9:xmax]);
ymax=1.2;
ylim([0 ymax])
set(gca,'Ytick',[])
set(gca,'Ytick',[0:ymax/6:ymax]);
xlabel('days','fontname','arial','fontsize',20);
ylabel('coefficients of Rt','fontname','arial','fontsize',20);
set(gca,'fontsize',20);
grid on
box off

% Analyze Tree，divided by the number of infected individuals ~100, 100~1000, 1000~10000, 10000~
mean_pr=proportion*chr';
%~100
Tree_1=Tree(find(Tree(:,6)<100),:);                   % Select rows from Tree with infection numbers ranging from 100 to 100
R0_1=Tree_1(:,2);
lambda_1=Tree_1(:,3);
hr_1=Tree_1(:,4);
t_inf1=Tree_1(:,5);
num_inf1=Tree_1(:,6);
%100~1000
Tree_2=Tree(find(100<=Tree(:,6) & Tree(:,6)<1000),:);   % Select rows with 100-1000 infected individuals from the Tree
```

```
R0_2=Tree_2(:,2);
lambda_2=Tree_2(:,3);
hr_2=Tree_2(:,4);
t_inf2=Tree_2(:,5);
num_inf2=Tree_2(:,6);
%1000~10000
Tree_3=Tree(find(1000<=Tree(:,6) & Tree(:,6)<10000),:);  % Select rows from the Tree with less than 100 infected individuals
R0_3=Tree_3(:,2);
lambda_3=Tree_3(:,3);
hr_3=Tree_3(:,4);
t_inf3=Tree_3(:,5);
num_inf3=Tree_3(:,6);
%10000~
Tree_4=Tree(find(Tree(:,6)>=10000),:);  % Select rows from the Tree with less than 100 infected individuals
R0_4=Tree_4(:,2);
lambda_4=Tree_4(:,3);
hr_4=Tree_4(:,4);
t_inf4=Tree_4(:,5);
num_inf4=Tree_4(:,6);

% Analyze the number of infected individuals and use R
m1=sum(Tree_1(:,6))/sum(Tree(:,6))*ones(length(Tree_1(:,1)),1);
m2=sum(Tree_2(:,6))/sum(Tree(:,6))*ones(length(Tree_2(:,1)),1);
m3=sum(Tree_3(:,6))/sum(Tree(:,6))*ones(length(Tree_3(:,1)),1);
m4=sum(Tree_4(:,6))/sum(Tree(:,6))*ones(length(Tree_4(:,1)),1);
M(:,1)=[m1;m2;m3;m4];
xlswrite('C:\Users\LENOVO\Desktop\M.xlsx',M)
library(ggplot2)
Tree0<-read.table("Tree1.txt",header=T)                        # Store Tree0 in the document
p1<-ggplot(Tree0,aes(label,value,color=label,fill=label))+
  geom_bar(stat="summary",fun=mean,position="dodge",width=0.5)+
  ylab("proportion")+
```

```
  xlab("number of infections")+
  theme(text = element_text(size = 18),
        axis.title = element_text(size = 20),
        axis.text.x = element_text(size = 14),
        axis.text.y = element_text(size = 18),
        legend.position="none")+
  geom_jitter(aes(y=value+0.2), position = position_jitterdodge(jitter.height = 0.2), color = "black",size=0.5)
  ggsave("p1.jpg",width=6,height=5)
  dev.off()

% Analye Rt,lambda,hr
%Rt
Rt0=Tree13(:,[2,6]);
xlswrite('C:\Users\LENOVO\Desktop\Rt0.xlsx',Rt0)
Rt0<-read.table("Rt0.txt",header=T)               # Store Tree0 in the document
install.packages("ggplot2")
library(ggplot2)
library(ggpubr)

Rt0<-read.table("Rt0.txt",header=T)
r1<-ggplot(Rt0,aes(label,value,fill=label))+
    geom_bar(stat="summary",fun=mean,position="dodge",width=0.5)+
    stat_summary(fun.data="mean_sd",geom="errorbar",width=0.3)+
    ylab("R0")+
    xlab("number of infections")+
    theme(text = element_text(size = 18),
        axis.title = element_text(size = 20),
        axis.text.x = element_text(size = 14),
        axis.text.y = element_text(size = 18),
        legend.position="none")+
        geom_signif(comparisons = list(c("10000~","~100"),
                                       c("10000~","100~1000"),
```

```
                                        c("1000~10000","~100"),
                                        c("1000~10000","100~1000")), # Set the groups that need to be compared
                    map_signif_level = T,                            # Whether to use asterisks to display
                    test = "t.test",                                 # method of calculation
                    y_position = c(5.2,5.6,6.0,6.4),                 # Setting the position of the horizontal line in the picture
                    tip_length = c(c(0.01,0.01),
                                   c(0.01,0.01),
                                   c(0.01,0.01),
                                   c(0.01,0.01)),                    # Vertical line setting below horizontal line
                    size=0.8,color="black")
ggsave("r1.jpg",width=6,height=5)
r1
dev.off()

%lambda
Lambda=Tree13(:,[3,6]);
xlswrite('C:\Users\LENOVO\Desktop\Lambda.xlsx',Lambda)

Lambda<-read.table("Lambda.txt",header=T)
la1<-ggplot(Lambda,aes(label,value,fill=label))+
    geom_bar(stat="summary",fun=mean,position="dodge",width=0.5)+
    stat_summary(fun.data="mean_sd",geom="errorbar",width=0.3)+
    ylab("lambda")+
    xlab("number of infections")+
    theme(text = element_text(size = 18),
        axis.title = element_text(size = 20),
        axis.text.x = element_text(size = 14),
        axis.text.y = element_text(size = 18),
        legend.position="none")+
        geom_signif(comparisons = list(c("10000~","~100"),
                                        c("10000~","100~1000"),
                                        c("1000~10000","~100"),
```

```
                                        c("1000~10000","100~1000")),     # Set the groups that need to be compared
                    map_signif_level = T,                                # Whether to use asterisks to display
                    test = "t.test",                                     # method of calculation
                    y_position = c(1.6,1.7,1.8,1.9),              # Setting the position of the horizontal line in the picture
                    tip_length = c(c(0.01,0.01),
                                   c(0.01,0.01),
                                   c(0.01,0.01),
                                   c(0.01,0.01)),                 # Vertical line setting below horizontal line
                    size=0.8,color="black")
ggsave("la1.jpg",width=6,height=5)
la1
dev.off()

%hr
mean_pr=proportion*chr';
t4=Tree13(:,4);
t4=t4*mean_pr;
Hr=[t4,Tree13(:,6)];
xlswrite('C:\Users\LENOVO\Desktop\Hr.xlsx',Hr)

Hr<-read.table("Hr.txt",header=T)
h1<-ggplot(Hr,aes(label,value,fill=label))+
    geom_bar(stat="summary",fun=mean,position="dodge",width=0.5)+
    stat_summary(fun.data="mean_sd",geom="errorbar",width=0.3)+
    ylab("hospitalization rate")+
    xlab("number of infections")+
    theme(text = element_text(size = 18),
        axis.title = element_text(size = 20),
        axis.text.x = element_text(size = 14),
        axis.text.y = element_text(size = 18),
        legend.position="none")+
        geom_signif(comparisons = list(c("10000~","~100"),
```

```
                                        c("10000~","100~1000"),
                                        c("1000~10000","~100"),
                                        c("1000~10000","100~1000")),
                    map_signif_level = T,
                    test = "t.test",
                    y_position = c(0.12,0.13,0.14,0.15),
                    tip_length = c(c(0.01,0.01),
                                   c(0.01,0.01),
                                   c(0.01,0.01),
                                   c(0.01,0.01)),
                    size=0.8,color="black")
ggsave("h1.jpg",width=6,height=5)
h1
dev.off()

%t
library(ggplot2)
library(ggpubr)
T<-read.table("T.txt",header=T)
t1<-ggplot(T,aes(label,value,fill=label))+
    geom_bar(stat="summary",fun=mean,position="dodge",width=0.5)+
    stat_summary(fun.data="mean_sd",geom="errorbar",width=0.3)+
    ylab("t")+
    xlab("number of infections")+
    theme(text = element_text(size = 18),
        axis.title = element_text(size = 20),
        axis.text.x = element_text(size = 14),
        axis.text.y = element_text(size = 18),
        legend.position="none")+
        geom_signif(comparisons = list(c("10000~","~100"),
                                        c("10000~","100~1000"),
                                        c("1000~10000","~100"),
```

```
                                        c("1000~10000","100~1000")),
                    map_signif_level = T,
                    test = "t.test",
                    y_position = c(330,360,390,420),
                    tip_length = c(c(0.01,0.01),
                                   c(0.01,0.01),
                                   c(0.01,0.01),
                                   c(0.01,0.01)),
                    size=0.8,color="black")
ggsave("t1.jpg",width=6,height=5)
t1
dev.off()

% Chain of transmission
D=xlsread('C:\Users\LENOVO\Desktop\D1.xlsx');
%Data
D0=D;
Data=[];
while length(D0(:,1))>0
    f1=find(D0(:,1)==D0(1,1));
    D1=D0(f1,:);
    data=[D0(1,1),max(D1(:,4))];
    Data=[Data;data];
    D0(f1,:)=[];
end
Link=D(:,[2,1]);
xlswrite('C:\Users\LENOVO\Desktop\Data.xlsx',Data)
xlswrite('C:\Users\LENOVO\Desktop\Link.xlsx',Link)

% Immune escape lambda
%lambda=[0.6,1.6];                           % The range of values for immune escape index lambda, positively correlated
n=300;
```

```
dis=[0:0.1:8];
lambda0=rand(1,n)+0.6;
hold on
Q=[];
for j=1:n
    lambda_1=lambda0(j);
    p_inf=1-exp(-lambda_1*dis);         % Probability of being infected under distance
    p=plot(dis,p_inf,'r')
    p.Color(4) = 0.05;
    for k=1:5
        Q(j,k)=1-exp(-lambda_1*k);
    end
end
lambda_1=median(lambda0);
p_inf=1-exp(-lambda_1*dis);              % Probability of being infected under distance
plot(dis,p_inf,'b','LineWidth',1)

for k=1:5
    q1=quantile(Q(:,k),[0.25,0.75]);
    xk=ones(1,100)*k;
    yk=linspace(q1(1),q1(2),100);
    plot(xk,yk,'b','LineWidth',1)
    xk0=linspace(k-0.05,k+0.05,10);
    yk_up=ones(1,10)*q1(2);
    yk_dw=ones(1,10)*q1(1);
    plot(xk0,yk_up,'b','LineWidth',1)
    plot(xk0,yk_dw,'b','LineWidth',1)
end
xmax=8;
xlim([0 xmax])
set(gca,'Xtick',[])
set(gca,'Xtick',[0:xmax/8:xmax]);
```

```
ymax=1.2;
ylim([0 ymax])
set(gca,'Ytick',[])
set(gca,'Ytick',[0:ymax/6:ymax]);
xlabel('genetic distance','fontname','arial','fontsize',20);
ylabel('probability of infection','fontname','arial','fontsize',20);
set(gca,'fontsize',20);
grid on
box off

%Par_t
%gamma_dow
n=500;
p_bot=0.3;                                  % The percentage of R0 decreasing to its maximum value
gamma_dow=0.2;                              % R0 decline rate, gamma low negatively correlated with R0
gamma_up=0.3;                               % R0 rise rate, gamma up is positively correlated with R0
par_max=rand(1,n)*(1-0.5)+0.5;
t=linspace(0,30,100);
Q=[];
hold on
for j=1:n
    par_max1=par_max(j);
    par_dow=(par_max1-p_bot)*2./(exp(-gamma_dow*t)+exp(gamma_dow*t))+p_bot;
    p=plot(t,par_dow,'m')
    p.Color(4)=0.05;
    q=[];
    for k=5:5:15
        par_dow0=(par_max1-p_bot)*2./(exp(-gamma_dow*k)+exp(gamma_dow*k))+p_bot;
        q=[q,par_dow0];
    end
    Q=[Q;q];
end
```

```
par_max1=median(par_max);
par_dow1=(par_max1-p_bot)*2./(exp(-gamma_dow*t)+exp(gamma_dow*t))+p_bot;
plot(t,par_dow1,'b','LineWidth',1)
for k=1:3
    q1=quantile(Q(:,k),[0.25,0.75]);
    xk=ones(1,100)*k*5;
    yk=linspace(q1(1),q1(2),100);
    plot(xk,yk,'b','LineWidth',1)
    xk0=linspace(5*k-0.2,5*k+0.2,10);
    yk_up=ones(1,10)*q1(2);
    yk_dw=ones(1,10)*q1(1);
    plot(xk0,yk_up,'b','LineWidth',1)
    plot(xk0,yk_dw,'b','LineWidth',1)
end
xmax=30;
xlim([0 xmax])
set(gca,'Xtick',[])
set(gca,'Xtick',[0:xmax/6:xmax]);
ymax=1;
ylim([0 ymax])
set(gca,'Ytick',[])
set(gca,'Ytick',[0:ymax/5:ymax]);
xlabel('days','fontname','arial','fontsize',20);
ylabel('values of \it\beta_1','fontname','arial','fontsize',20);
set(gca,'fontsize',20);
grid on
box off

%gamma_up
par_min=rand(1,n)*(0.6-0.3)+0.3;
t=linspace(0,30,100);
Q=[];
```

```
hold on
for j=1:n
    par_min1=par_min(j);
    par_up=1-2*(1-par_min1)./(exp(-gamma_up*t)+exp(gamma_up*t));
    p=plot(t,par_up,'Color',[0.9290 0.6940 0.1250])
    p.Color(4)=0.05;
    q=[];
    for k=5:5:15
        par_up0=1-2*(1-par_min1)./(exp(-gamma_up*k)+exp(gamma_up*k));
        q=[q,par_up0];
    end
    Q=[Q;q];
end
par_min1=median(par_min);
par_up1=1-2*(1-par_min1)./(exp(-gamma_up*t)+exp(gamma_up*t));
plot(t,par_up1,'b','LineWidth',1)

for k=1:3
    q1=quantile(Q(:,k),[0.25,0.75]);
    xk=ones(1,100)*k*5;
    yk=linspace(q1(1),q1(2),100);
    plot(xk,yk,'b','LineWidth',1)
    xk0=linspace(5*k-0.2,5*k+0.2,10);
    yk_up=ones(1,10)*q1(2);
    yk_dw=ones(1,10)*q1(1);
    plot(xk0,yk_up,'b','LineWidth',1)
    plot(xk0,yk_dw,'b','LineWidth',1)
end
xmax=30;
xlim([0 xmax])
set(gca,'Xtick',[])
set(gca,'Xtick',[0:xmax/6:xmax]);
```

```
ymax=1;
ylim([0 ymax])
set(gca,'Ytick',[])
set(gca,'Ytick',[0:ymax/5:ymax]);
xlabel('days','fontname','arial','fontsize',20);
ylabel('values of \it\beta_2','fontname','arial','fontsize',20);
set(gca,'fontsize',20);
grid on
box off

% New_inf with 10 cycles
New_inf=xlsread('C:\Users\LENOVO\Desktop\New_inf.xlsx');
[rN,cN]=size(New_inf);
if cN<450
    New_inf=[New_inf,zeros(10,450-cN)];
end
t=1:450;
x=[t,flip(t)];
New_inf75=prctile(New_inf,75);
New_inf50=prctile(New_inf,50);
New_inf25=prctile(New_inf,25);
E1=[New_inf75,flip(New_inf25)];
f1=fill(x, E1, 'b','facealpha',0.4,'edgealpha',0);
hold on
plot(t,New_inf50,'b','LineWidth',1)
legend('interquartile range','the median','FontSize',20,'box','off')
xmax=450;
xlim([0 xmax])
set(gca,'Xtick',[])
set(gca,'Xtick',[0:xmax/9:xmax]);
ymax=1800;
ylim([0 ymax])
```

```
set(gca,'Ytick',[])
set(gca,'Ytick',[0:ymax/9:ymax]);
xlabel('days','fontname','arial','fontsize',20);
ylabel('no. of new individuals','fontname','arial','fontsize',20);
set(gca,'fontsize',20);
grid on
box off
xlswrite('C:\Users\LENOVO\Desktop\New_inf.xlsx',New_inf)

% 10 cycles
Par=xlsread('C:\Users\LENOVO\Desktop\Par.xlsx');
% After the end of the epidemic, people's lives returned to normal and Par increased to 1
[rP,cP]=size(Par);
if cP<450
    Par=[Par,zeros(10,450-cP)];
end

for k=1:10                              % Par has a total of 10 rows
    par=[];
    fm=min(find(Par(k,:)==0));          % The end time of the epidemic
    par_min=Par(k,fm-1);                % The initial value of Par_min
    t0=fm-1;                            % The initial value of t0
    t_=1:450-t0;                        % T-value
    par=1-2*(1-par_min)./(exp(-gamma_up*t_)+exp(gamma_up*t_));
    Par(k,fm:450)=par;
end
t=1:450;
x=[t,flip(t)];
Par75=prctile(Par,75);
Par50=prctile(Par,50);
Par25=prctile(Par,25);
E1=[Par75,flip(Par25)];
```

```
f1=fill(x, E1, 'b','facealpha',0.4,'edgealpha',0);
hold on
plot(t,Par50,'-b','LineWidth',1)
legend('interquartile range','the median','FontSize',20,'box','off')
xmax=450;
xlim([0 xmax])
set(gca,'Xtick',[])
set(gca,'Xtick',[0:xmax/9:xmax]);
ymax=1.2;
ylim([0 ymax])
set(gca,'Ytick',[])
set(gca,'Ytick',[0:ymax/6:ymax]);
xlabel('days','fontname','arial','fontsize',20);
ylabel('coefficients of Rt','fontname','arial','fontsize',20);
set(gca,'fontsize',20);
grid on
box off
xlswrite('C:\Users\LENOVO\Desktop\Par.xlsx',Par)

% evolutionary tree
% establish Link
Tree=xlsread('C:\Users\LENOVO\Desktop\Tree.xlsx');
Tree0=Tree;
[cT,rT]=size(Tree0);
Link=[];
while cT>0
    if Tree0(1,1)==1000
        f1=find(1000<Tree0(:,1) & Tree0(:,1)<=1900);
    else
        f1=find(Tree0(1,1)*100<Tree0(:,1) & Tree0(:,1)<=Tree0(1,1)*100+99);
    end
    if length(f1)>0
```

```
        T1=[ones(length(f1),1)*Tree0(1,1),Tree0(f1,1)];
        Link=[Link;T1];
    end
    Tree0(1,:)=[];
    [cT,rT]=size(Tree0);
end
% Replace large numbers in Link
for j=1:length(Tree(:,1))
    f1=find(Link==Tree(j,1));
    Link(f1)=j;
end
xlswrite(’C:\Users\LENOVO\Desktop\Link0.xlsx’,Link)

% Establish Data
Data=[];
Data(:,1)=1:length(Tree(:,1));
Data(:,2)=Tree(:,6);
Data=sortrows(Data,-2);
xlswrite(’C:\Users\LENOVO\Desktop\Data0.xlsx’,Data)

% Epidemic time and number of infections
New_inf=xlsread(’C:\Users\LENOVO\Desktop\New_inf.xlsx’);
t=[];
persons=[];
for j=1:length(New_inf(:,1))
    t_max=max(find(New_inf(j,:)>0));
    t=[t;t_max];
    pj=sum(New_inf(j,:));
    persons=[persons;pj];
end
csvwrite(’C:\Users\LENOVO\Desktop\t.csv’,t)
csvwrite(’C:\Users\LENOVO\Desktop\persons.csv’,persons)
```

```
#R Violin diagram
library(ggplot2)
t<-read.csv("t.csv")
tp<-ggplot(t, aes(x = species, y = values, fill=species))+
      geom_violin(trim = F,fill="#FFCD00FF",width=0.5)+
      geom_boxplot(width=0.2)+
      geom_jitter(shape = 16, position = position_jitter(0.1),size =2,colour = "blue")+
      ylab("days")+
      xlab("")+
      ylim(c(0,600))+
      theme(text = element_text(size = 18),
            axis.title = element_text(size = 30),
            axis.text.x = element_text(size = 30),
            axis.text.y = element_text(size = 30))+
      guides(fill=F)
ggsave("t_0.001_0.3.pdf",width=4.2,height=5)
tp
dev.off()

persons<-read.csv("persons.csv")
pp<-ggplot(persons, aes(x = species, y = values, fill=species)) +
      geom_violin(trim = F,fill="#84BD00FF",width=0.5)+
      geom_boxplot(width=0.2)+
      geom_jitter(shape = 16, position = position_jitter(0.1),size = 2,colour = "blue")+
      ylab("number of people")+
      ylim(c(17800,18600))+
      xlab("")+
      theme(text = element_text(size = 18),
            axis.title = element_text(size = 30),
            axis.text.x = element_text(size = 30),
            axis.text.y = element_text(size = 30))+
```

```
        guides(fill=F)
ggsave("p_0.002_0.1.pdf",width=5,height=5)
pp
dev.off()
```
