# Supplementary material for: Quantitatively analyzing the relationship between non-pharmaceutical interventions and the direction of virus evolution using a dynamic model
Source: Front Public Health. 2025 May 9;13:1542759. doi: 10.3389/fpubh.2025.1542759 (PMC12098584; doi:10.3389/fpubh.2025.1542759)
Supplement: APPENDIX II — The sensitivity analysis of quantitatively analyzing the relationship between non-pharmaceutical interventions and the direction of virus evolution using a dynamic model. (DOI:10.17632/g67n3x7nj7.1). [file Data_Sheet_2.pdf]

```
%clear all
np=20000;
proportion=[0.276,0.284,0.292,0.148];
chr=[0.0033,0.0145,0.0331,0.0789];
na=np*proportion;
R0=[2,5];
lambda=[0.6,1.6];
hr=[0.5,5];
p_imm0=0.1;
mu_inc0=3.1;
sd_inc=2.6;
q0=0.002;
p0=0.1;
delta=0.0467;
gamma_dow0=0.2;
gamma_up0=0.3;
p_bot0=0.25;
thr_hos0=0.05;
unthr_hos0=0.04;
name=1000;
ss=20;
nc=9;

Ro=[]; Infections=[];
for tc=8:nc
    Ran_p_imm=randsample([1:ss],ss);
    p_imm=p_imm0*0.9+(Ran_p_imm-1)*p_imm0*0.2/ss+p_imm0*0.2/ss*rand;

    Ran_mu_inc=randsample([1:ss],ss);
    mu_inc=mu_inc0*0.9+(Ran_mu_inc-1)*mu_inc0*0.2/ss+mu_inc0*0.2/ss*rand;

    Ran_q=randsample([1:ss],ss);
```

```
q=q0*0.9+(Ran_q-1)*q0*0.2/ss+q0*0.2/ss*rand;

Ran_p=randsample([1:ss],ss);
p=p0*0.9+(Ran_p-1)*p0*0.2/ss+p0*0.2/ss*rand;

Ran_gamma_dow=randsample([1:ss],ss);
gamma_dow=gamma_dow0*0.9+(Ran_gamma_dow-1)*gamma_dow0*0.2/ss+gamma_dow0*0.2/ss*rand;

Ran_gamma_up=randsample([1:ss],ss);
gamma_up=gamma_up0*0.9+(Ran_gamma_up-1)*gamma_up0*0.2/ss+gamma_up0*0.2/ss*rand;

Ran_p_bot=randsample([1:ss],ss);
p_bot=p_bot0*0.9+(Ran_p_bot-1)*p_bot0*0.2/ss+p_bot0*0.2/ss*rand;

Ran_thr_hos=randsample([1:ss],ss);
thr_hos=thr_hos0*0.9+(Ran_thr_hos-1)*thr_hos0*0.2/ss+thr_hos0*0.2/ss*rand;

Ran_unthr_hos=randsample([1:ss],ss);
unthr_hos=unthr_hos0*0.9+(Ran_unthr_hos-1)*unthr_hos0*0.2/ss+unthr_hos0*0.2/ss*rand;

Ranks=[];
Ranks(:,1)=Ran_p_imm'; Ranks(:,2)=Ran_mu_inc';Ranks(:,3)=Ran_q'; Ranks(:,4)=Ran_p';
Ranks(:,5)=Ran_gamma_dow'; Ranks(:,6)=Ran_gamma_up';Ranks(:,7)=Ran_p_bot'; Ranks(:,8)=Ran_thr_hos';
Ranks(:,9)=Ran_unthr_hos';

% Start a big loop
infections=[];
for j=1:ss
    hos_Inf=[];                                  % Store hospitalization rates for newly infected individuals
    % Establish a population file Fp, with each row representing one person. Each column includes: 1 age, 2 congenital immunity, 3 number of previous infections, 4 type of strain
    Fp=zeros(np,4);
    % The first column is divided into four age groups
```

```
c1=[];
for i=1:length(na)
    c1=[c1;ones(round(na(i)),1)*i];
end
Fp(:,1)=c1;

% 10% of the second column has innate immunity, represented by 1
r1=randperm(np);
id_imm=r1(1:round(p_imm(j)*np));            % Congenital immune person id_imm
id_sus=1:np;
id_sus(id_imm)=[];                           % Vulnerable person id_sus
Fp(id_imm,2)=1;

% Initially, there were n0 strains, R0_1, lambda_1, hr_1, and they were stored in the Tree
 name0=name;
R0_1=5;
lambda_1=median(lambda);
hr_1=median(hr);

new_inf0=0;
while new_inf0<=100
    par_T=[];
    D=[];
    Tree=[];
    Tree(:,1)=name0;Tree(:,2)=R0_1;Tree(:,3)=lambda_1;Tree(:,4)=hr_1;Tree(:,5)=0;
    E=[];
    % First case id_1
    r_sus=randperm(length(id_sus));
    id_1=id_sus(r_sus(1));
    id_sus(r_sus(1))=[];

    D(1,1)=id_1; D(1,2)=0; D(1,3)=name; D(1,4)=1; D(1,5)=Fp(id_1,1);
```

```
D(1,7)=0; D(1,8)=0; D(1,12)=1; D(1,13)=0;
% Determine whether to be hospitalized
age=Fp(id_1,1);
hr_2=chr(age)*hr_1;
fh=binornd(1,hr_2);
% Infection period and length of hospital stay
if fh==1
    per_inf=rand*(12-4)+4;
    per_hos=rand*(20-12)+12;
    % Judging whether there is death
    dr=binornd(1,delta);
    if dr==1
        D(1,6)=2;
    else
        D(1,6)=1;
    end
    D(1,9)=per_inf;
    D(1,10)=D(1,9);
    D(1,11)=D(1,10)+per_hos;
else
    D(1,6)=0;
    D(1,9)=rand*(7-2)+2;
    D(1,10)=0;
    D(1,11)=D(1,9);
end

% Update the Fp of the first case, 3 previous infections, 4 strain types
Fp(id_1,3)=1;
Fp(id_1,4)=D(1,3);
nu_inf=poissrnd(Tree(1,2));
D(1,13)=1;
```

```
if nu_inf>0
    times_inf=rand(1,nu_inf)*D(1,9);
    E0=[ones(1,nu_inf)*D(1,1);times_inf;ones(1,nu_inf)*name];
    E=[E,E0];
end

[rE,cE]=size(E);
if cE>0
    t=min(E(2,:));
    index_t=find(E(2,:)==t);
    e=E(:,index_t);
    E(:,index_t)=[];
    t1=0;
    t2=0;
    Par_t=[];
    Par_t(:,1)=[1;0];
    tt=1;
    New_Tre=[];
    id_rec=[];
end

% Start small loop
while cE>0 & t<=tc*50
    f1=find(D(:,11)<t & D(:,12)==1 & D(:,6)~=2);
    D(f1,12)=2;
    id_rec=[id_rec,D(f1,1)'];
    f2=find(D(:,11)<=t & D(:,12)==1 & D(:,6)==2);
    D(f2,12)=3;
    id_inf=D(find(D(:,12)==1),1)';
    id_hos=D(find(D(:,6)~=0 & D(:,10)<t & t<=D(:,11)),1)';

    % Selecting the next generation of infected individuals can only be done from id_sus, id-rec, id-inf, and id_imm
```

```
id_cum=[id_sus,id_rec,id_inf,id_imm];
r_next=round(rand*length(id_cum)+0.5);
% Determine whether transmission will occur
id_sr=[id_sus,id_rec];
strain0=e(3);

if r_next<=length(id_sr)
    % Determine whether the strain has mutated
    r_var=binornd(1,q(j));
    fT=find(Tree(:,1)==strain0);

    if r_var==1
        % Determine the name of the new strain
        if mod(max(Tree(:,1)),10)==0
            b=ceil(log10(max(Tree(:,1))))+1;
        else
            b=ceil(log10(max(Tree(:,1))));
        end
        Tree0=num2str(Tree(:,1),b);
        Tree0(find(Tree0==' '))='0';
        s_T=size(Tree0);
        % The number of digits in strin0
        if mod(strain0,10)==0
            b0=ceil(log10(strain0))+1;
        else
            b0=ceil(log10(strain0));
        end
        strain0=num2str(strain0,b0);
        if str2num(strain0)==name
            f_1000=find(Tree(:,1)<name*2);
            strain_1=max(Tree(f_1000,1))+1;
        else
```

```
    family=[];
    k=1;
    while k<=s_T(1)
        f0=min(find(Tree0(k,:)>'0'));
        f1=f0-1+length(strain0);
        if f1<=s_T(2) & str2num(Tree0(k,f0:f1))==str2num(strain0)
            family=[family;str2num(Tree0(k,f0:s_T(2)))];
        end
        k=k+1;
    end
    f2=find(str2num(strain0)<=family & family<=str2num(strain0)*name/10+(name/10-1));
    family1=family(f2);

    if length(family1)==1
        strain_1=str2num(strain0)*name/10+1;
    else
        strain_1=max(family1)+1;
    end
end

% Determine the new R0, lambda, and hr, and the mutation strategy is to mutate all three indicators,
 % with a variation amplitude exceeding the original p-fold
Ranges=[R0;lambda;hr];
pres=Tree(fT,2:4);
news=[];
for j0=1:3
    val_new=rand*(Ranges(j0,2)-Ranges(j0,1))+Ranges(j0,1);
    val_pre=pres(j0);
    while abs(val_new-val_pre)/val_pre<p(j)
        val_new=rand*(Ranges(j0,2)-Ranges(j0,1))+Ranges(j0,1);
    end
    news(j0)=val_new;
```

```
    end
    % Store the information of the new strain in a tree
    tree=[];
    tree(1:4)=[news,t];
    tree=[strain_1,tree];
    Tree=[Tree;tree];
    R0_1=tree(2);
    lambda_1=tree(3);
    hr_1=tree(4);
else
    strain_1=strain0;
    R0_1=Tree(fT,2);
    lambda_1=Tree(fT,3);
    hr_1=Tree(fT,4);
end

% Determine the type of person in contact (sus or rec)
distance=[];
if r_next>length(id_sus)
    % Determine whether the rehabilitation patient will be reinfected
    % Find the infected strain of the recovered individual
    state0=2;
    id_infected=id_sr(r_next);
    f_r=find(D(:,1)==id_infected & D(:,12)==2);
    type=D(f_r,3);
    type=unique(type);
    Types0=[strain_1;type];
    % Calculate the genetic distance between the infectious source strain and the infected person strain
    % Disassemble the strains of Types and store the gene loci of each strain in each column of Strains
     if mod(max(Types0),10)==0
        b=ceil(log10(max(Types0)))+1;
    else
```

```
    b=ceil(log10(max(Types0)));
end
Types=num2str(Types0,b);
Types(find(Types==' '))='0';
Strains=[];
size_T=size(Types);
for k=1:size_T(1)
    k0=1;
    c_min=min(find(Types(k,:)>'0'));
    Strains(k,k0)=str2num(Types(k,c_min));
    for k1=1:floor((size_T(2)-c_min)/2)
        k0=k0+1;
        if k0==2
            Strains(k,k0)=str2num(Types(k,c_min+[1:3]));
        else
            Strains(k,k0)=str2num(Types(k,c_min+1+[1:2]+2*(k1-1)));
        end
    end
end

for k=2:size_T(1)
    if Strains(1,:)==Strains(k,:)
        distance0=0;
    else
        k0=1;
        while Strains(1,k0)==Strains(k,k0)
            k0=k0+1;
        end
        if Strains(1,k0)==0 | Strains(k,k0)==0
            distance0=abs(max(find(Strains(1,:)>0))-max(find(Strains(k,:)>0)));
        else
            d1=max(find(Strains(1,:)>0))-(k0-1);
```

```
                d2=max(find(Strains(k,:)>0))-(k0-1);
                distance0=d1+d2;
            end
        end
        distance(k-1)=distance0;
    end
else
    state0=1;
    id_infected=id_sr(r_next);
    distance=1000;
end

% Calculate the probability of infection under distance
p_inf=1-exp(-lambda_1*distance);
p_0=prod(p_inf);
inf0=binornd(1,p_0);
if inf0==1
    d=[];
    d(1)=id_infected;
    d(2)=e(1);
    d(3)=strain_1;
    % Determine the number of infections
    d(4)=length(find(D(:,1)==d(1)))+1;
    % Update Fp, id_sus, id_rec
    Fp(d(1),3)=d(4);
    Fp(d(1),3+d(4))=d(3);
    if state0==1
        id_sus(find(id_sus==d(1)))=[];
    else
        id_rec(find(id_rec==d(1)))=[];
    end
    d(5)=Fp(d(1),1);
```

```
d(7)=e(2);
% Incubation period
inc_per=normrnd(mu_inc(j),sd_inc);
while inc_per<1 | inc_per>5
    inc_per=normrnd(mu_inc(j),sd_inc);
end
d(8)=inc_per;
% Whether hospitalized or deceased
hr_2=chr(d(5))*hr_1;
fh=binornd(1,hr_2);
% Infection period and length of hospital stay
if fh==1
    % Judging whether there is death
    dr=binornd(1,delta);
    if dr==1
        d(6)=2;
    else
        d(6)=1;
    end
    d(9)=rand*(12-4)+4;
    d(10)=d(7)+d(8)+d(9);
    per_hos=rand*(20-12)+12;
    d(11)=d(10)+per_hos;
else
    d(6)=0;
    d(9)=rand*(7-2)+2;
    d(10)=0;
    d(11)=d(7)+d(8)+d(9);
end
d(12)=1;
d(13)=0;
D=[D;d];
```

```
    end
end

 % Determine the number of effective contacts of all infectious sources at time t during the infection period
 % First, determine the coefficient par-R0 of R0 at time t
 % Determine the hospitalization rate of newly infected individuals at time t. If the hospitalization rate exceeds
 % Thr_hos, an alert will be issued
 day=floor(t);
new_hos=length(find(D(:,6)~=0 & day-1<=D(:,10) & D(:,10)<day));
new_inf=length(find(day-1<=D(:,7) & D(:,7)<day));
if new_inf>0
    hos_Inf=[hos_Inf,new_hos/new_inf];
else
    hos_Inf=[hos_Inf,0];
end
r_min=max(length(hos_Inf)-4,1);
hos_inf=mean(hos_Inf(r_min:length(hos_Inf)));

if t1==0 & t2==0 & hos_inf>=thr_hos(j)
    t1=t;
elseif t1==0 & t2==0 & hos_inf<thr_hos(j)
    par_t=[1;t];
    par_max=1;
end
if t1>0 & t2==0 & hos_inf>=unthr_hos(j)
    % If a warning has been issued and the hospitalization rate is greater than the warning release value
    t_=t-t1;
    par_R0=(par_max-p_bot(j))*2./(exp(-gamma_dow(j)*t_)+exp(gamma_dow(j)*t_))+p_bot(j);
    par_t=[par_R0;t];
elseif t1>0 & t2==0 & hos_inf<unthr_hos(j)
    % If a warning has been issued and the hospitalization rate reaches the warning lifting value
    t_=t-t1;
```

```
    par_min=(par_max-p_bot(j))*2./(exp(-gamma_dow(j)*t_)+exp(gamma_dow(j)*t_))+p_bot(j);
    par_t=[par_min;t];
    t1=0;
    t2=t;
elseif t2>0 & t1==0 & hos_inf<thr_hos(j)
    % If the warning is lifted and the hospitalization rate is less than the warning value
    t_=t-t2;
    par_R0=1-2*(1-par_min)/(exp(-gamma_up(j)*t_)+exp(gamma_up(j)*t_));
    par_t=[par_R0;t];
elseif t2>0 & t1==0 & hos_inf>=thr_hos(j)
    % If the number of inpatients reaches the warning value again after the warning is lifted
    t_=t-t2;
    par_max=1-2*(1-par_min)/(exp(-gamma_up(j)*t_)+exp(gamma_up(j)*t_));
    par_t=[par_max;t];
    t2=0;
    t1=t;
end
Par_t=[Par_t,par_t];

r_inf=find(D(:,7)+D(:,8)<=t & t<=D(:,7)+D(:,8)+D(:,9) & D(:,13)==0);
D(r_inf,13)=1;
id_infectors=D(r_inf,1);
for j0=1:length(id_infectors)
    %Find the strain var0 and its R0_var0 of the infectious source
    var0=D(r_inf(j0),3);
    R0_var0=Tree(find(Tree(:,1)==var0),2);
    id_dea=D(find(D(:,12)==3),1);
    p_1=(length(id_hos)+length(id_dea))/np;
    p_Rt=Par_t(1,find(Par_t(2,:)==t));
    p_Rt=(1-p_1)*p_Rt;
    Rt=R0_var0*p_Rt;
    nu_inf=poissrnd(Rt);
```

```
                if nu_inf>0
                    time_inf=rand(1,nu_inf)*D(r_inf(j0),9)+D(r_inf(j0),7)+D(r_inf(j0),8);
                    Time_inf=[ones(1,nu_inf)*id_infectors(j0);time_inf;ones(1,nu_inf)*D(r_inf(j0),3)];
                    E=[E,Time_inf];
                end
            end
            t=min(E(2,:));
            index_t=find(E(2,:)==t);
            e=E(:,index_t);
            E(:,index_t)=[];
            [rE,cE]=size(E);

            % Display program progress by day
            if fix(t)+1~=tt
                tt=fix(t)+1;
                [tc,j,tt]
            end
        end
        new_inf0=length(D(:,1));
    end
    infections(j)=new_inf0;
end

% The serial number of per_inf
[i1,i2]=sort(infections);
[i3,i4]=sort(i2);
Ran_r=i4';

% correlation coefficient
for r=1:9
    XX1=[];
    for iii=1:9
```

```
            if iii~=r
                XX1(:,iii)=Ranks(:,iii);
            else
                XX1(:,iii)=ones(ss,1);
            end
        end
        XX2=XX1*inv(XX1'*XX1)*XX1'*Ranks(:,r);
        YY2=XX1*inv(XX1'*XX1)*XX1'*Ran_r;
        Xz=Ranks(:,r)-XX2;
        Yz=Ran_r-YY2;
        Ro(r,tc)=dot(Xz,Yz)/norm(Xz)/norm(Yz);
    end
    Ro
    Infections=[Infections,infections'];
    xlswrite('C:\Users\LENOVO\Desktop\Ro.xlsx',Ro)
    xlswrite('C:\Users\LENOVO\Desktop\New_inf.xlsx',Infections)
end

%PRCC
ro=xlsread('C:\Users\LENOVO\Desktop\Ro.xlsx');
hold on
t=[1:9];
k=5;
M3=ro(3,:);
P=polyfit(t,M3,k);
yy=polyval(P,t);
p1=plot(t,yy,'-r','LineWidth',1.5,'MarkerSize',10)

M4=ro(4,:);
P=polyfit(t,M4,k);
yy=polyval(P,t);
p3=plot(t,yy,'-g','LineWidth',1.5,'MarkerSize',10)
```

```
M6=ro(6,:);
P=polyfit(t,M6,k);
yy=polyval(P,t);
p4=plot(t,yy,'-k','LineWidth',1.5,'MarkerSize',10)

M5=ro(5,:);
P=polyfit(t,M5,k);
yy=polyval(P,t);
p5=plot(t,yy,'-','Color','#ffbb00','LineWidth',1.5,'MarkerSize',10)

tt=1:0.1:9;
y=zeros(1,length(tt));
plot(tt,y,'--k','LineWidth',1.5)

axis([1,9,-1,1])
set(gca,'Xtick',[1:9]);
set(gca,'XtickLabel',{'50';'100';'150';'200';'250';'300';'350';'400';'450'},'fontsize',22,'fontname','arial');
xlabel('days','fontsize',26,'fontname','arial')
ylabel('PRCC','fontsize',26,'fontname','arial')
%p_antitest=0.1, int_test=2, lambda=2, mu_inc=3.1, mu_pos=8.4, p_antibody=0.1, sensitivity=0.87
l1=legend('\fontsize{22}\itq','\fontsize{20}\itp','\fontsize{22}\it\gamma_{up}','\fontsize{22}\it\gamma_{dow}','Location','NorthEast')
set(l1,'Box','off','Orientation','horizon')
set(gca, 'Ytick', [-1:0.2:1])
grid on

hold on
M1=ro(1,:);
P=polyfit(t,M1,k);
yy=polyval(P,t);
p6=plot(t,yy,'-r','LineWidth',1.5,'MarkerSize',10)
```

```
M7=ro(7,:);
P=polyfit(t,M7,k);
yy=polyval(P,t);
p7=plot(t,yy,'-g','LineWidth',1.5,'MarkerSize',10)

M8=ro(8,:);
P=polyfit(t,M8,k);
yy=polyval(P,t);
p8=plot(t,yy,'-k','LineWidth',1.5,'MarkerSize',10)

M9=ro(9,:);
P=polyfit(t,M9,k);
yy=polyval(P,t);
p9=plot(t,yy,'-','Color','#ffbb00','LineWidth',1.5,'MarkerSize',10)

tt=1:0.1:9;
y=zeros(1,length(tt));
plot(tt,y,'--k','LineWidth',1.5)

axis([1,9,-1,1])
set(gca,'Xtick',[1:9]);
set(gca,'XtickLabel',{'50';'100';'150';'200';'250';'300';'350';'400';'450'},'fontsize',22,'fontname','arial');
xlabel('days','fontsize',26,'fontname','arial')
ylabel('PRCC','fontsize',26,'fontname','arial')
%p_antitest=0.1, int_test=2, lambda=2, mu_inc=3.1, mu_pos=8.4, p_antibody=0.1, sensitivity=0.87
legend('\fontsize{22}\itp_{imm}','\fontsize{22}\itp_{bot}','\fontsize{22}\itupper bound','\fontsize{22}\itlower bound','Location','NorthEast')
set('Box','off','Orientation','horizon')
set(gca, 'Ytick', [-1:0.2:1])
grid on
```
